# Die shift prediction of fan out panel level packages considering both warpage and flow induced mechanisms with temperature dependent properties

**DOI:** 10.1038/s41598-025-34076-2

**Published:** 2026-01-03

**Authors:** Yu-Chi Sung, Chih-Ping Hu, Chun-Chieh Hung, Sheng-Jye Hwang, Ming-Hsien Shih, Wen-Hsiang Liao, Yong-Jie Zeng, Cheng-Tse Tsai

**Affiliations:** 1 Department of Mechanical Engineering, National Cheng Kung University, Tainan, 70101 Taiwan; 2 Department of Mechanical Engineering, Southern Taiwan University of Science and Technology, Tainan, 710301 Taiwan; 3 Packaging Product Simulation and Design, Innolux Corporation, Tainan, 744092 Taiwan; 4 Testing Center, Innolux Corporation, Tainan, 744092 Taiwan

**Keywords:** Fan-out panel-level packaging, Compression molding, Die shift, Nanoindentation, Mold tape, Warpage, Engineering, Materials science, Mathematics and computing, Physics

## Abstract

Fan-out panel-level packaging is a leading technology in advanced semiconductor manufacturing, yet the phenomenon of die shift during the compression molding process remains a significant challenge to yield and reliability. This study presents a comprehensive investigation into the mechanisms of die shift, categorizing them into warpage-induced and fluid-flow-induced effects. By integrating material characterization, advanced finite element modeling, and nanoindentation analysis, we evaluate the thermal softening behavior of heat release tape and its contribution to die shift. It is found that the Young’s modulus of heat release tape (HRT) decreases by a factor of 2 to 3 at elevated temperatures, substantially increasing fluid-induced die shift. Simulations incorporating this high-temperature behavior improve agreement with experimental measurements by up to 48%. Moreover, results show that warpage effects dominate die shift in peripheral die locations, while fluid-flow effects intensify with local die density. This work establishes a validated multiphysics framework for accurate die shift prediction, highlighting the critical role of temperature-dependent mechanical properties in modeling real-world packaging scenarios.

## Introduction

With the rapid advancement of technology, the demand for 3 C products has been steadily increasing. In line with the ongoing trend toward high-density integration and miniaturized semiconductor packages, fan-out panel-level packaging (FOPLP) has emerged as a promising solution. By utilizing large panels, FOPLP improves cost efficiency and packaging throughput by addressing edge-area waste associated with wafer-level packaging.

**Fig. 1:**
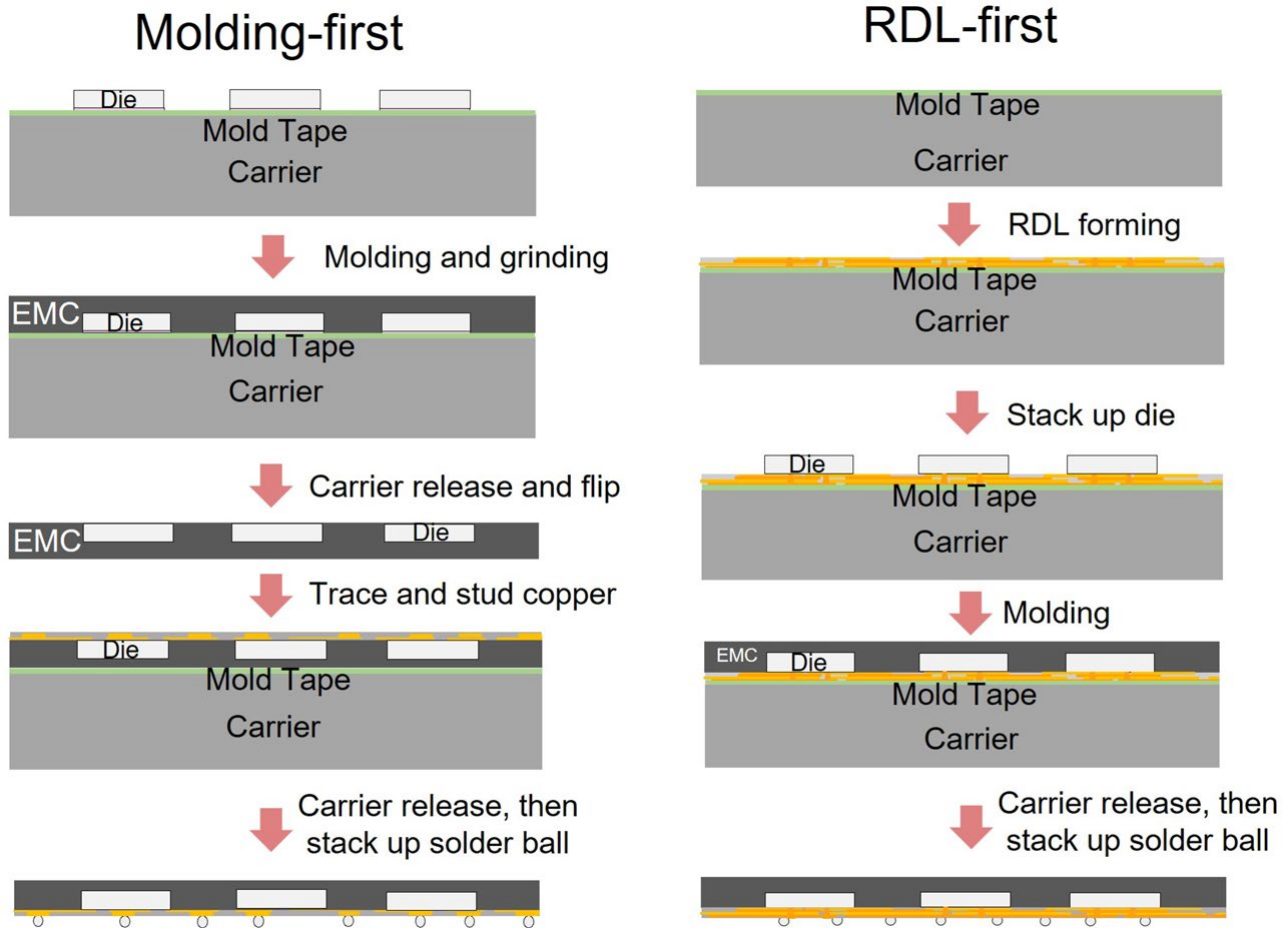
Process flow chart of molding-first and RDL-first.

FOPLP technologies are primarily categorized into two approaches: the redistribution layer (RDL)-first process and the molding-first process^1,2^. In the RDL-first approach, dies and RDL layers are assembled on the carrier prior to encapsulation and carrier removal. In contrast, the molding-first process begins with molding dies onto the carrier, followed by a grinding step for surface planarization, carrier removal, and subsequent copper layer deposition beneath the die^3^. This study focuses on the molding-first process, and the process flows for both approaches are illustrated in Fig. 1.

During the initial molding stage, die shift often occurs due to resin drag flow and cure shrinkage. This deformation can result in misalignment during the RDL process and compromise product quality. Previous studies have attributed the main causes of die shift to two key mechanisms: fluid flow effects resulting from non-uniform mold filling and warpage effects induced by epoxy molding compound (EMC) shrinkage and thermal expansion mismatch among different materials^4,5^.

Three general deformation directions can be observed in die shift: inward, outward, and random. In general, warpage-induced die shift tends to move outward toward the panel center due to non-uniform thermal contraction, whereas flow-induced displacement exhibits a more random or flow-direction-dependent behavior.

Several investigations have explored these mechanisms and contributing factors. Khong and Lin demonstrated that excessive compression speeds generate extremely high peak drag forces, leading to more severe die shift, especially near the panel edges^4,6^. Ling investigated the adhesive properties of mold tapes under varying processing temperatures and found that die shift is significantly influenced by the degree of tape adhesion^7^. In addition, as a thermosetting polymer, EMC undergoes volume changes during the curing process. Hwang analyzed EMC chemical shrinkage using differential scanning calorimetry (DSC) and developed a pressure-volume-temperature-conversion (*P-V-T-C*) model to describe the relationship between shrinkage and cure rate under isothermal and isobaric conditions^8^. Subsequent studies by Hwang and Chang further confirmed that processing temperature plays a critical role in affecting both the cure reaction rate and the extent of shrinkage^9,10^. Hong, Deng, and Hwang extended this work by integrating finite element analysis with the *P-V-T-C* model to simulate mold flow and predict warpage behavior^11,12^. These findings validated the effectiveness of incorporating thermomechanical relationships into molding simulations. Li and Yu investigated die fly and die shift in fan-out wafer-level packaging using coupled mold-flow and curing simulations. They successfully eliminated die fly and limited die shift within 50 μm across a wafer of nearly 5000 dies. Their findings highlight the strong coupling between resin flow, curing dynamics, and material properties during molding^13^.

Despite these advancements, most existing simulations neglect the temperature-dependent mechanical softening of HRT, which can significantly influence die behavior during molding. This study addresses this limitation by employing nanoindentation experiments to characterize the Young’s modulus of HRT at elevated temperatures. The resulting temperature-dependent properties are integrated into the simulation model to improve the accuracy of die shift prediction. This approach provides enhanced physical insight into the distinct roles of warpage and fluid flow across different die locations and establishes a validated framework for realistic die shift evaluation in FOPLP.

## Mold flow analysis and fluid-structure interaction theorem

### Mold flow analysis

A filling analysis was conducted to simulate the distribution of the melting front, molding temperature, velocity, and conversion within the cavity. Fluid flow phenomena are described by the continuity equation, momentum equation, and energy equation.

#### Continuity equation

1 $$\frac{{\partial \rho }}{{\partial {\rm{t}}}}\;{\rm{ + }}\;\nabla{\cdot} \left( {\rho \mathop v\limits^ \to } \right)\;{\rm{ = 0}}$$

$\rho$: density; *t*: time; $\:\stackrel{{\rightarrow}}{\mathrm{v}}$: velocity vector; $\:{\nabla}\cdot$: divergence operator.

#### Momentum equation

2 $$\:\rho \left( {\frac{{\partial \mathop {\rm{V}}\limits^ \to }}{{\partial {\rm{t}}}}{\rm{ + }}\mathop {\rm{V}}\limits^ \to \cdot \;\nabla \mathop {\rm{V}}\limits^ \to } \right)\:{\rm{ = }}\;\nabla\: \cdot {\mathop \sigma \limits^ = _{total}}{\rm{ + }}\:\rho \mathop g\limits^ \to$$

3 $${\mathop \sigma \limits^ = {_{total}}}{\rm{ = - }}\mathop \sigma \limits^ = {\rm{ + }}\:\mathop \tau \limits^ =$$

∇: gradient operator; ${\mathop \sigma \limits^ = {_{total}}}$: total stress tensor; *P*: pressure, *P* ${\mathop \delta \limits^ =}$: hydrostatic stress; ${\mathop \tau \limits^ =}$: extra stress; $\:\stackrel{{\rightarrow}}{\mathrm{g}}$: gravity.

#### Energy equation

4 $$\rho {c_p}\left( {\frac{{\partial {\rm{t}}}}{{\partial {\rm{t}}}}{\rm{ + }}\mathop {\rm{v}}\limits^ \to \cdot \;\nabla {\rm{t}}} \right)\:{\rm{ = }}\:\;\nabla \cdot ({\rm{k}}\;\nabla {\rm{t}}){\rm{ + }}\:(\mathop {\tau \:}\limits^ - :\;\nabla \mathop {\rm{v}}\limits^ \to ){\rm{ + }}\:\mathop {\rm{a}}\limits^. \Delta {\rm{h}}$$

$\:\mathrm{c}\mathrm{p}$: specific heat of EMC; *t*: temperature; $\:\mathrm{k}$: heat conductivity of EMC; $\:\dot{{\alpha}}$: cure rate of EMC; $\:{\Delta}{h}$: enthalpy change.

### Fluid-structure interaction analysis

Fluid-structure interaction (FSI) numerical simulation analysis primarily employs the fluid dynamics momentum equation and the solid mechanics elasticity equation. It is assumed that the materials obey Generalized Hooke’s law, and the die shift calculations are described using the force equilibrium Eqs^14,15^.

5 $$\:\nabla \cdot \mathop \sigma \limits^ = {\rm{ + }}\:\mathop {\rm{F}}\limits^ \to {\rm{ = 0}}$$

6 $$\:\mathop \sigma \limits^ ={\rm{ = }}\mathop {\rm{K}} \limits^ = {\rm}(\mathop \varepsilon \limits^ = - \mathop {{{\mathop \varepsilon \limits^ = }^0}}\limits^{} - {\mathop \alpha \limits^ = {_{{\rm{CLTE}}}}}\Delta {\rm{T}})$$

7 $$\:\mathop \epsilon \limits^ ={\rm{ = }}\frac{1}{2}(\nabla\bar{U}+\nabla\bar{U}^{T})$$

${\mathop \sigma \limits^ =}$: Stress tensor of die shift analysis; $\:\stackrel{{\rightarrow}}{\mathrm{F}}$: Force, primarily caused by the drag force of EMC; ${\mathop {{\rm}K} \limits^ =}$: 4th tensor; ${\mathop \epsilon \limits^ =}$: Total strain tensor; ${\mathop \epsilon \limits^ = }\mathrm{0}$: Initial strain tensor; ${\mathop \sigma \limits^ =}_\mathrm{CLTE}$: Coefficient of thermal expansion tensor; $\:\mathrm{T}$: Temperature; $\:\stackrel{\mathrm{-}}{\mathrm{U}}$: Displacement vector.

### Material models

#### Viscosity model

**Fig. 2:**
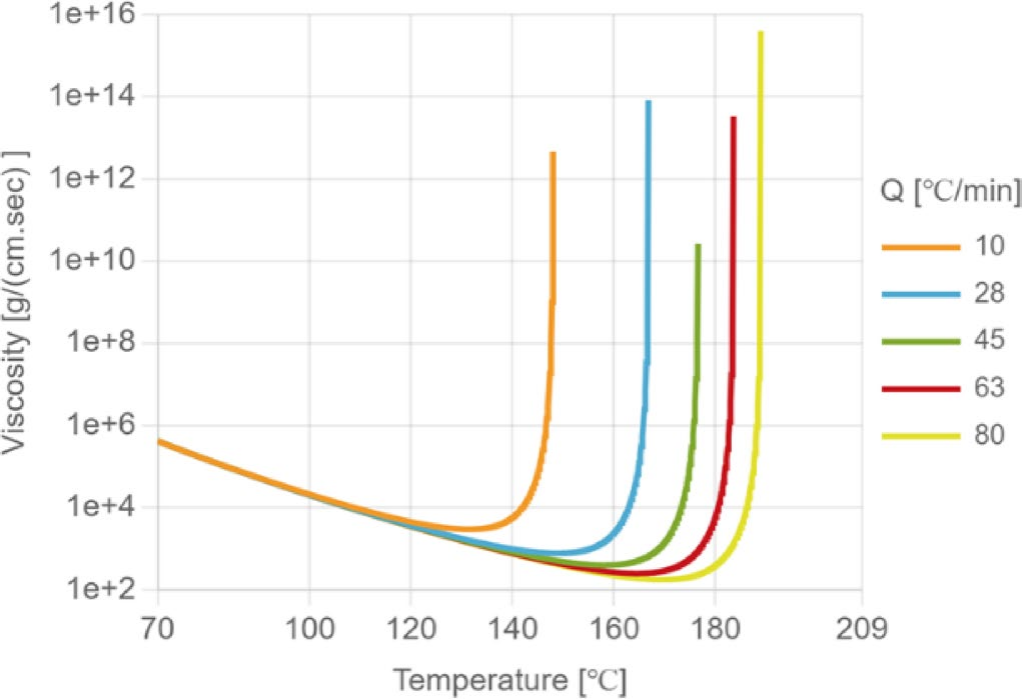
The relationship between viscosity and temperature under different heating rates.

Cross Castro-Macosko model^16^ is used to describe the viscosity behavior of EMC, considering the effects of curing, temperature, and shear strain rate on viscosity, which indirectly affect the filling process. The relationship between viscosity and temperature is graphically illustrated in Fig. 2, demonstrating the variation in viscosity with different temperatures.

As shown in Fig. 2, the viscosity of the EMC decreases with increasing temperature at the initial heating stage due to thermal softening. However, when the temperature approaches the curing region, a sudden increase in viscosity is observed. This abrupt rise corresponds to the onset of crosslinking reactions, where the EMC gradually transitions from a viscous liquid to a gel-like state. The heating rate also has a pronounced influence on the viscosity–temperature behavior. At lower heating rates, the resin has more time to react, and the crosslinking process begins at a lower temperature, leading to an earlier increase in viscosity. In contrast, at higher heating rates, the reaction is thermally delayed, and the rise of viscosity occurs at higher temperatures. The steeper viscosity increase observed under faster heating indicates a more rapid liquid-to-gel transition, reflecting the strong time–temperature coupling in the curing kinetics.

8 $$\eta {\rm{ = }}\:\frac{{\eta {\rm{0}}{{\left( {\frac{{{\rm{Cg}}}}{{{\rm{Cg - C}}}}} \right)}^{{\rm{K1 + K2C}}}}}}{{{\rm{1 + }}{{\left( {\frac{{\eta {\rm{0}}\mathop \gamma \limits^. }}{{{{\rm{t}}^{\rm{*}}}}}} \right)}^{{\rm{10n}}}}}}$$

9 $${\eta _0} = A \cdot exp(\frac{{{\rm{T}}b}}{{\rm{T}}})\:{\rm{Tb}}\:{\rm{ = }}\:\frac{{{\rm{E}}\eta }}{{\rm{R}}}$$

$\:\eta$: viscosity; $\:{\eta}_{\mathrm{0}}$: zero-shear-rate viscosity; $\:\mathrm{C}\mathrm{g}$: degree of cure at gel point; $\:\mathrm{C}$: degree of cure; $\:\dot{{\gamma}}$: shear strain rate; $\:{\tau\:}^{{*}}$: critical shear stress; $\:\mathrm{n}$: power law index; *K*_1_, *K*_2_, *A* and $\:\mathrm{T}\mathrm{b}$: model parameters; $\:\mathrm{E}\eta$: activation energy; $\:\mathrm{R}$: ideal gas constant.

#### Cure kinetics model

**Fig. 3:**
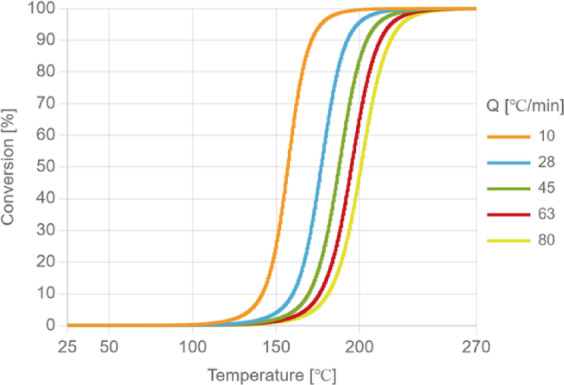
The heat absorption curve of the material as temperature increases under different rates of temperature increase.

Kamal’s model^17^ is used to describe the effect of temperature and time on the degree of cure of EMC material. The relationship between conversion rate and temperature is shown in Fig. 3.

10 $$\:\mathop {\rm{C}}\limits^. {\rm{ = }}\:\frac{{{\rm{dC}}}}{{{\rm{dt}}}}{\rm{ = (k1 + k2Cm)\cdot(1 - C)n}}$$

11 $$\:\mathrm{k}\mathrm{1}\text{}\mathrm{=\:}\mathrm{A}\mathrm{1}\text{}\mathrm{(}\frac{\mathrm{-}\mathrm{T}\mathrm{A}}{\mathrm{T}}\mathrm{)}$$

12 $$\:\mathrm{k}\mathrm{2}\mathrm{=\:}\mathrm{A}\mathrm{2}\text{}\mathrm{(}\frac{\mathrm{-}\mathrm{T}\mathrm{B}}{\mathrm{T}}\mathrm{)}$$

$\:\mathrm{k}\mathrm{1}$, $\:\mathrm{k}\mathrm{2}$: Rate constant of the cure reaction, which follows the Arrhenius equation; *m*, *n*, $\:\mathrm{A}\mathrm{1}$, $\:\mathrm{A}\mathrm{2}$: Model constants; *T*_*A*_, *T*_*B*_: Activation temperature; *T*: Temperature.

#### P-V-T-C model

**Fig. 4:**
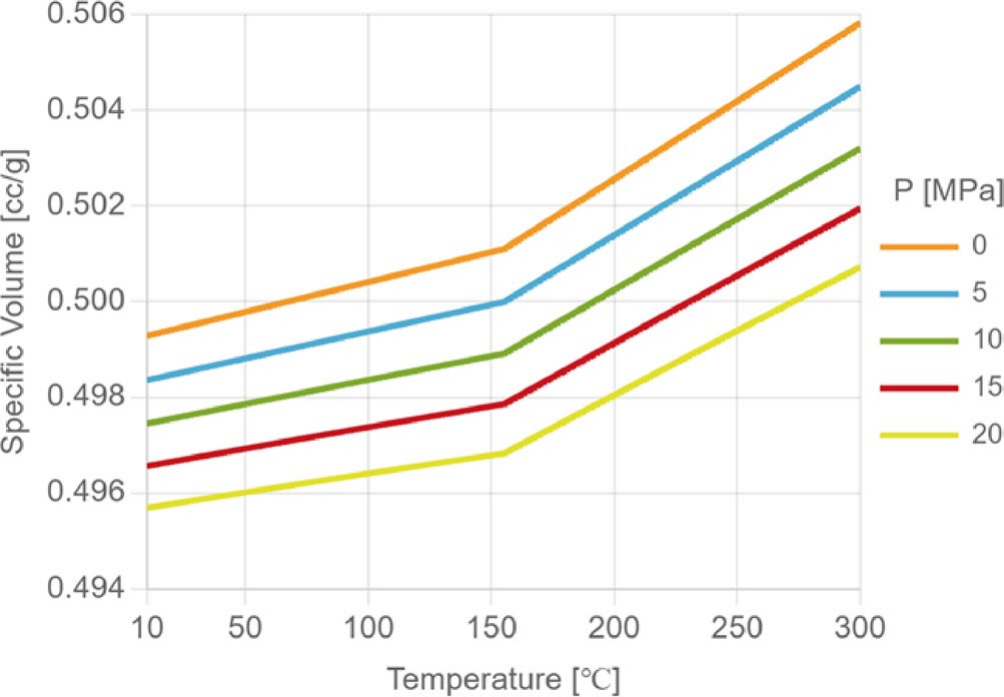
*P*-*V*-*T*-*C* relationship.

Two domain modified Tait model^8^ is used to describe the relation between *P*-*V*-*T*-*C* behavior of EMC in this study. *P*-*V*-*T*-*C* model characterizes the relationship between specific volume and degree of cure of EMC under different pressure and temperature conditions. The *P*-*V*-*T*-*C* relationship is shown in Fig. 4.

13 $$\:\frac{\mathrm{1}}{\mathrm{V}}\text{}\mathrm{=\:}\frac{\mathrm{1}}{\mathrm{V}\mathrm{uncured}}\cdot\left(\mathrm{1-}\mathrm{C}\right)\mathrm{+\:}\frac{\mathrm{1}}{\mathrm{V}\mathrm{cured}}\cdot\mathrm{C}$$

14 $$\:\mathrm{V}\mathrm{uncured}\mathrm{/cured}\text{}\mathrm{=\:}\mathrm{V}\mathrm{0}\mathrm{[1-}{\alpha}{\cdot}\mathrm{ln(1+}\frac{\mathrm{P}}{\mathrm{B}}\mathrm{)]}$$

15 $${\rm{V0 = }}\:\left\{ {\begin{array}{*{20}{c}} {{\rm{b1s + b2s}}\left( {{\rm{T - b5}}} \right){\rm{,ifT = Ttrans}}}\\ {\:{\rm{b1L + b2L}}\left( {{\rm{T - b5}}} \right){\rm{,ifT}}> {\rm{Ttrans}}} \end{array}} \right.$$

$\:\mathrm{V}$: Specific volume; *C*: Degree of cure; $\:\mathrm{V}\mathrm{0}$: Specific volume at zero gauge pressure; $\:\mathrm{P}$: Pressure; *B*: Accounts for pressure sensitivity of the material; *b*_1*s*_, *b*_2*s*_, *b*_1*L*_, *b*_2*L*_, *b*_3*s*_, *b*_4*s*_, *b*_3*L*_, *b*_4*L*_: Material constants; $\:\mathrm{b}\mathrm{5}$: Transition temperature at zero gauge pressure; $\:\mathrm{b}\mathrm{6}$: Linear increase in *T*_*trans*_ with pressure.

**Fig. 5:**
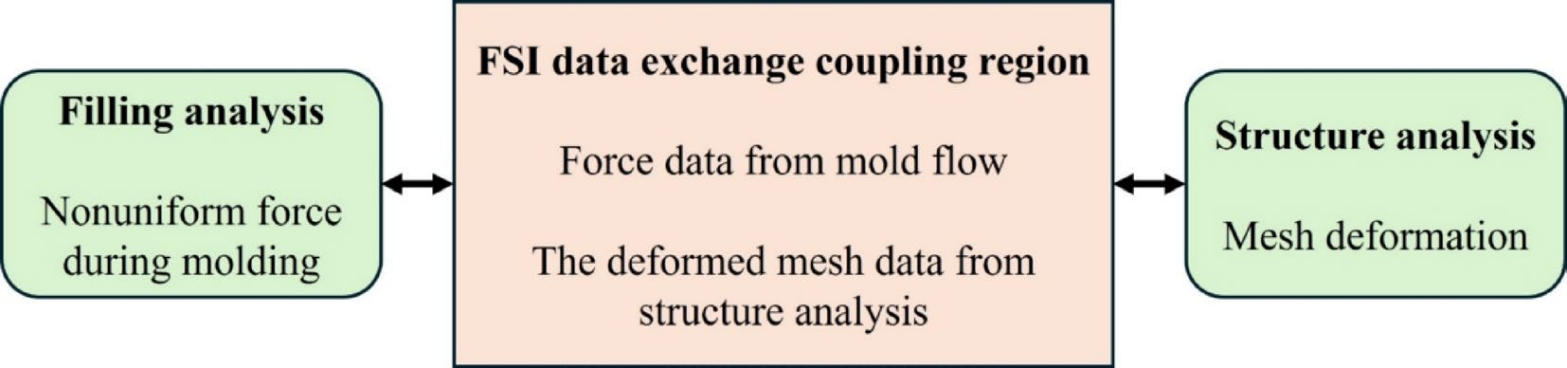
Calculation flow of two-way FSI filling analysis.

**Fig. 6:**
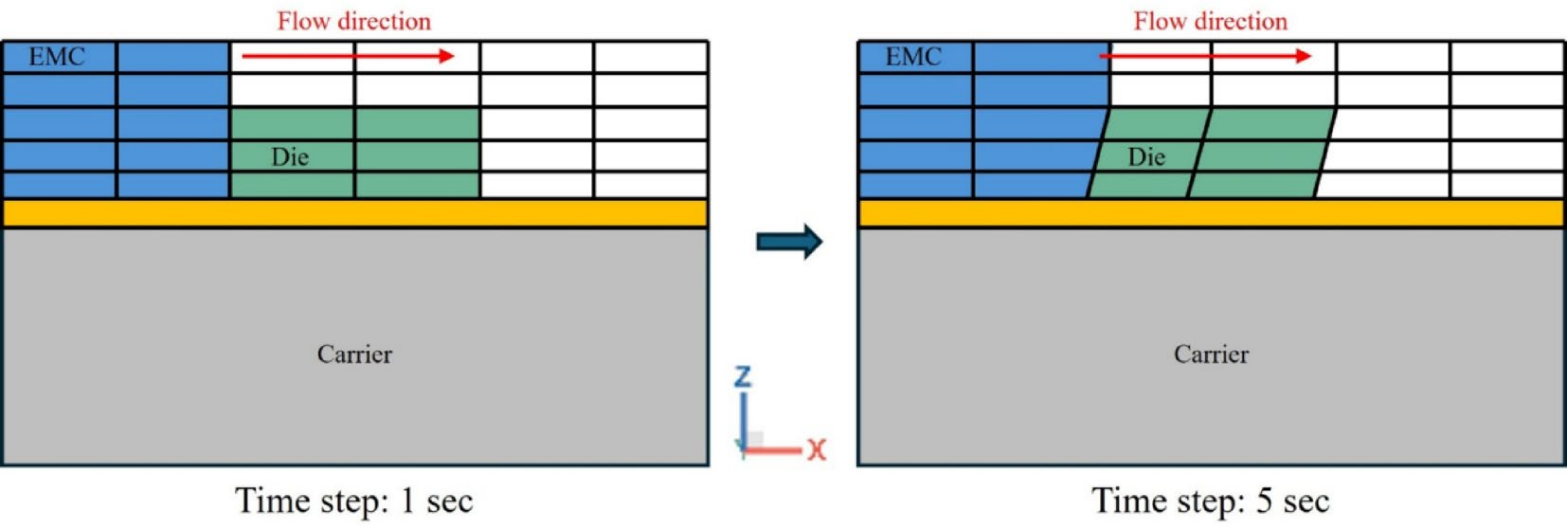
Schematic of mesh deformation behavior.

In this study, the die shift was analyzed by classifying its origins into two primary mechanisms: fluid-flow effects and warpage-induced effects. The fluid-flow behavior was simulated using the two-way fluid-structure interaction (FSI) filling analysis module in Moldex3D, as illustrated in Figs. 5 and 6. This approach enables the simulation to capture the dynamic mechanical response of the die under non-uniform pressure fields generated during encapsulant flow. Specifically, the two-way FSI method allows for iterative coupling between the resin flow and structural deformation, where flow-induced forces deform the die, and the resulting mesh distortion is continuously updated and fed back into the flow field. Such coupling is essential for accurately predicting transient die displacement during the molding stage, which conventional one-way or decoupled simulations often overlook.

In parallel, the warpage-induced die shift was evaluated using the Warp Solver module in Moldex3D. This analysis was conducted after the filling process to simulate the thermomechanical behavior of the molded package as it cools to room temperature. The resulting warpage profiles provide insight into the deformation driven by thermal contraction and material property mismatch, further contributing to the overall die shift.

## Numerical simulations

In this study, die shift simulations were conducted using Moldex3D to examine the respective contributions of fluid flow and thermally induced warpage. The simulation framework was developed based on the molding-first process in fan-out panel-level packaging, with particular attention to how material definitions influence the prediction of die behavior. In conventional modeling practices, material properties such as those of the heat release tape are commonly defined at room temperature, despite the fact that actual compression molding is performed under elevated thermal conditions. To address this discrepancy, this section presents a series of simulations that consider room-temperature mechanical properties as the initial baseline. The analysis includes the geometric configuration of the package structure, the mesh strategy and boundary settings, the assigned material properties, and the simulation results associated with both warpage effects and flow-induced displacement.

### Die shift analysis with room temperature HRT properties

**Fig. 7:**
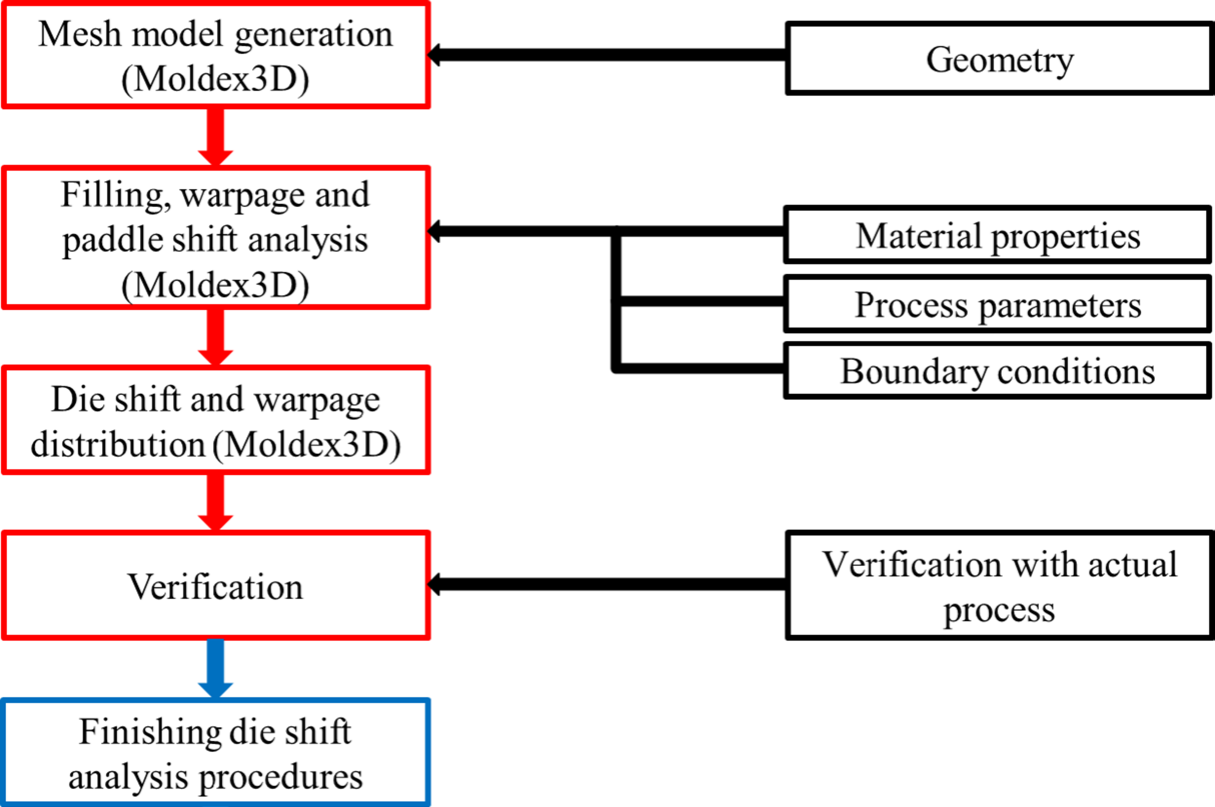
Analysis procedures of die shift analysis.

Heat release tape (HRT) is an adhesive tape with a plastic film base and a coated adhesive layer, widely used in the semiconductor industry for temporarily securing carriers. In this section, the HRT is modeled as a single thin layer, with only its room-temperature material properties considered in the simulation. Details on geometric dimensions, process parameters, boundary conditions, and material properties are also presented in this section. The procedure for die shift analysis is illustrated in Fig. 7.

#### Geometry

**Fig. 8:**
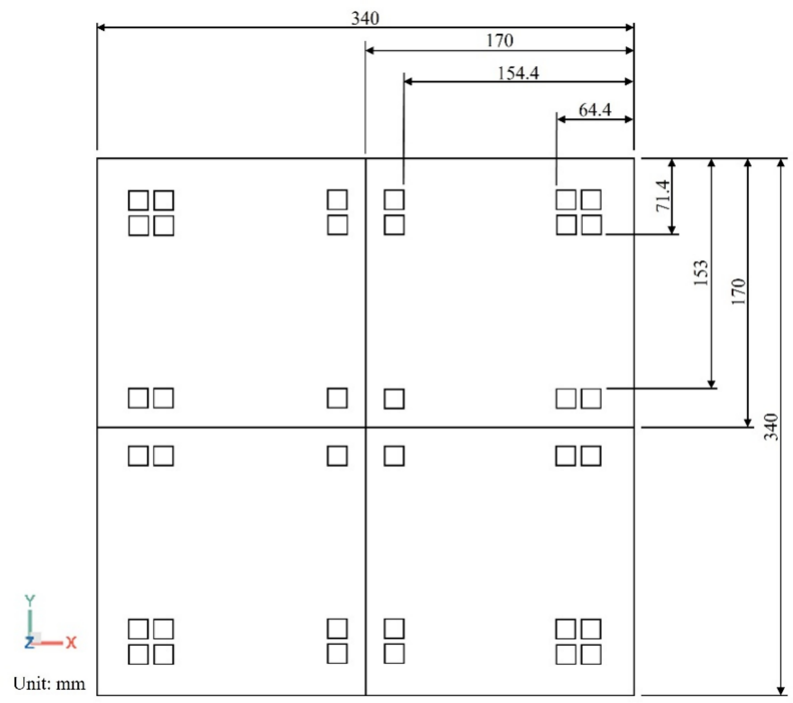
Top view of the geometry of full panel.

**Fig. 9:**
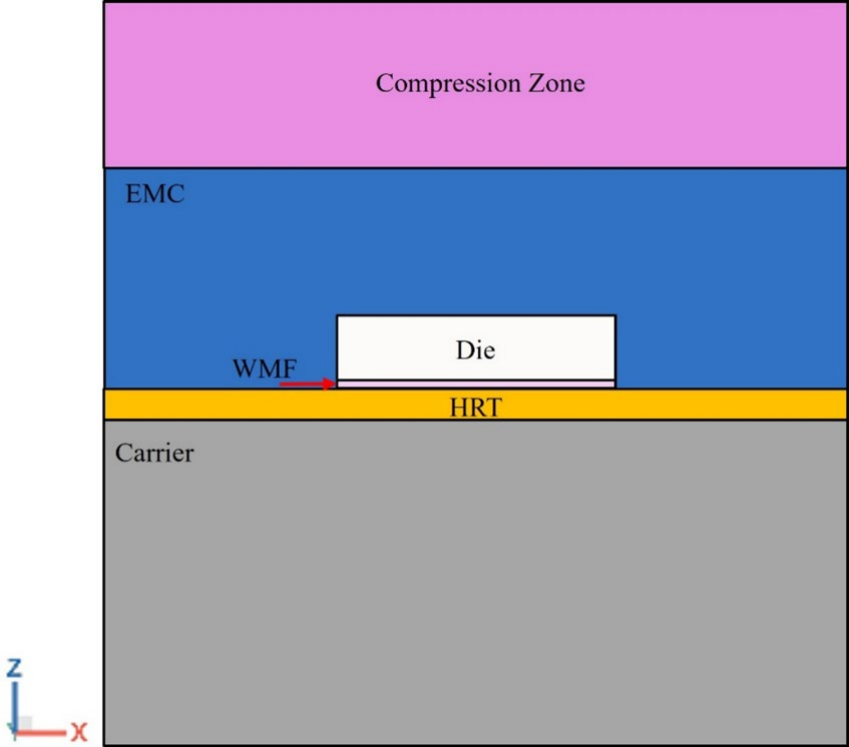
Schematic sectional view of the package model.

The geometric model used for die shift analysis, provided by InnoLux, is illustrated in Fig. 8. This model comprises four sub-panels, each containing nine dies. A schematic cross-sectional view of the package structure is shown in Fig. 9. The structural layers arranged from bottom to top, include a carrier, heat release tape (HRT), dielectric film (WMF), die, and epoxy molding compound (EMC). A compression zone was designated on the panel package model to simulate the compression molding process.

#### Mesh model and boundary conditions

**Fig. 10:**
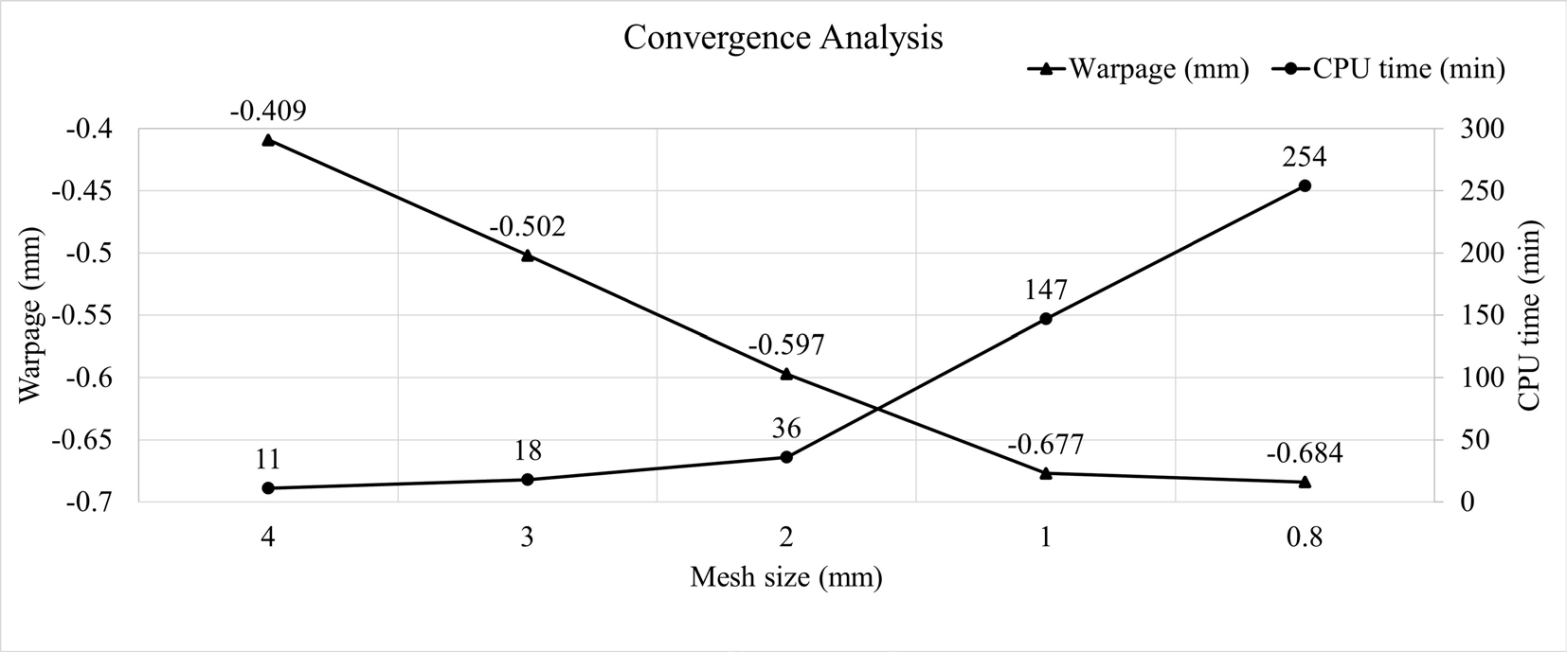
Results of convergence analysis.

To assess mesh sensitivity and ensure reliable simulation accuracy, five finite element models with different mesh sizes were constructed, as summarized in Table 1. The number of elements ranges from approximately 0.5 million for a 4 mm mesh to over 13 million for a 0.8 mm mesh. Figure 10 presents the results of the convergence analysis, highlighting the trade-off between warpage prediction and computational cost. As the mesh becomes finer, the warpage values gradually converge, with only a minimal change observed between the 1 mm and 0.8 mm cases. However, this marginal improvement is accompanied by a substantial increase in CPU time, rising from 147 min to 254 min. Based on this convergence behavior and the rapidly growing computational cost, a mesh size of 1 mm was selected for all subsequent simulations to ensure a balance between accuracy and efficiency.

**Table 1:** Number of mesh elements for different mesh sizes.

Mesh size	4 mm	3 mm	2 mm	1 mm	0.8 mm
Number of elements	519,322	949,292	2,118,168	8,413,834	13,010,717

**Fig. 11:**
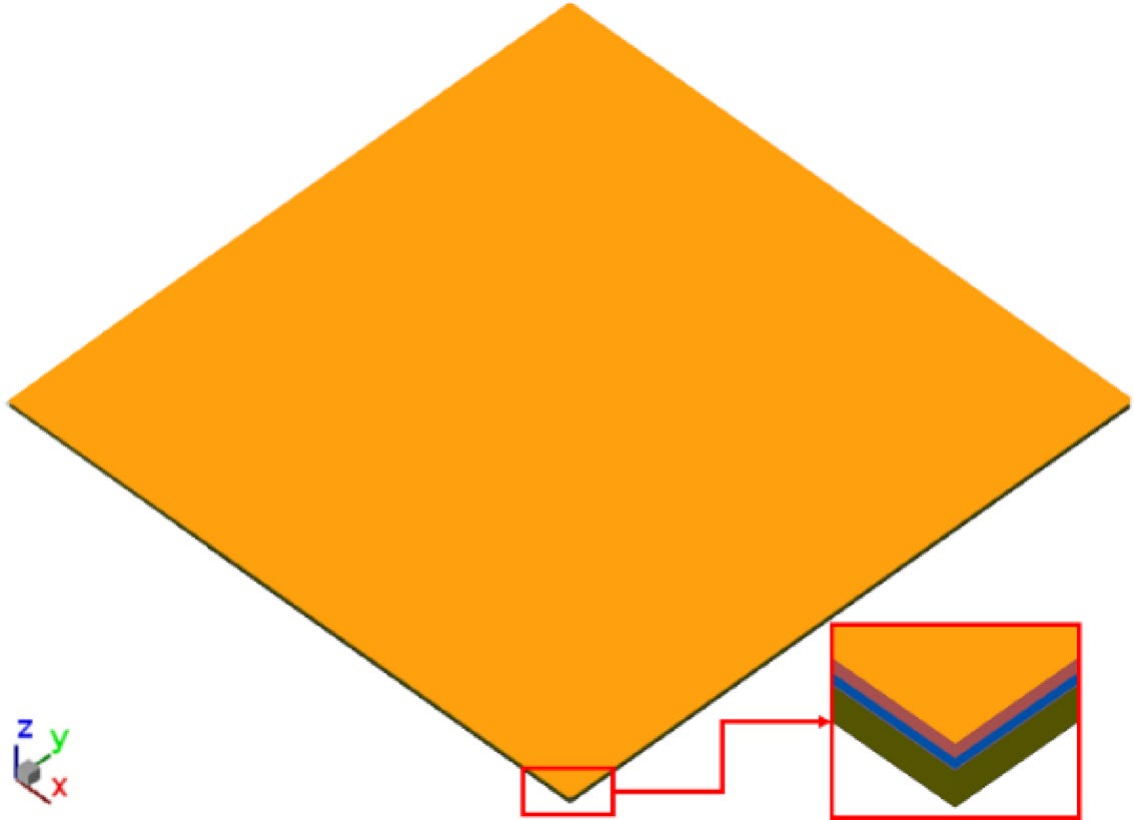
Boundary condition setting for moving surface.

**Fig. 12:**
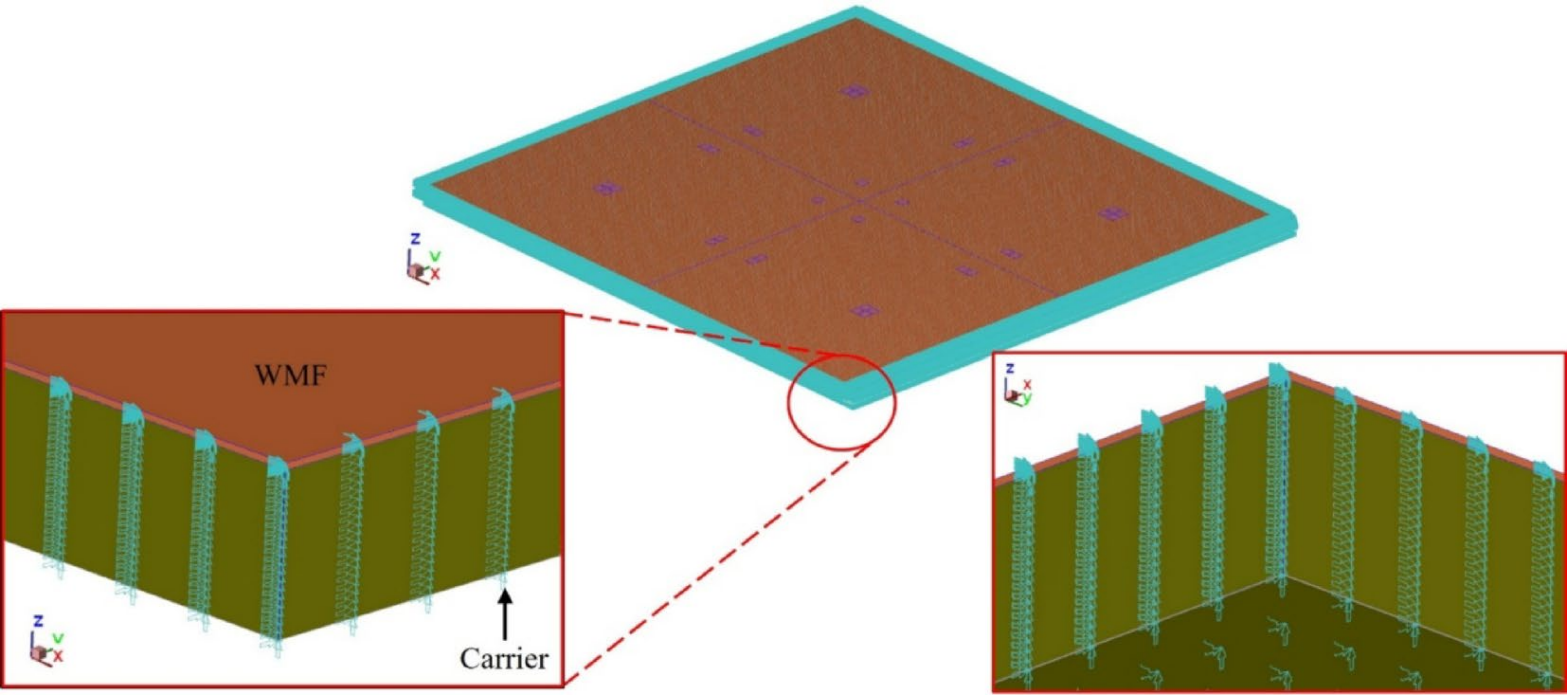
Boundary condition setting during die shift analysis induced by fluid-flow effects.

The boundary conditions were defined to capture the key physical mechanisms involved in compression molding and die shift. Gravitational acceleration was included in all simulations to account for weight-induced deformation. To simulate the compression molding process, a moving surface was applied above the designated compression zone, as shown in Fig. 11. For the fluid-flow induced die shift analysis, displacement constraints were imposed on the peripheral regions of the carrier and heat release tape to prevent rigid body motion, as illustrated in Fig. 12.

#### Material properties

The material properties used in this study were provided by Innolux Corporation and are specified at room temperature. For the epoxy molding compound, additional characterizations were performed by CoreTech, including the viscosity behavior, cure kinetics, and *P-V-T-C* relationships. These models enable a more accurate representation of EMC behavior during the molding process. A summary of all room-temperature material properties adopted in the simulations is listed in Table 2.

**Table 2:** Material properties used in the FEA.

Materias Properties	Carrier	HRT	WMF	Die	EMC
Young’s modulus (GPa)	210	0.108	9	131	25
Coefficient of thermal expansion (10^− 6^/˚C)	12	5	16	2.8	8.8
Poisson’s ratio	0.3	0.49	0.3	0.28	0.3
Density (g/cm^3^)	7.87	1.5	0.9	0.3	1.996

#### Die shift results with warpage effect

**Fig. 13:**
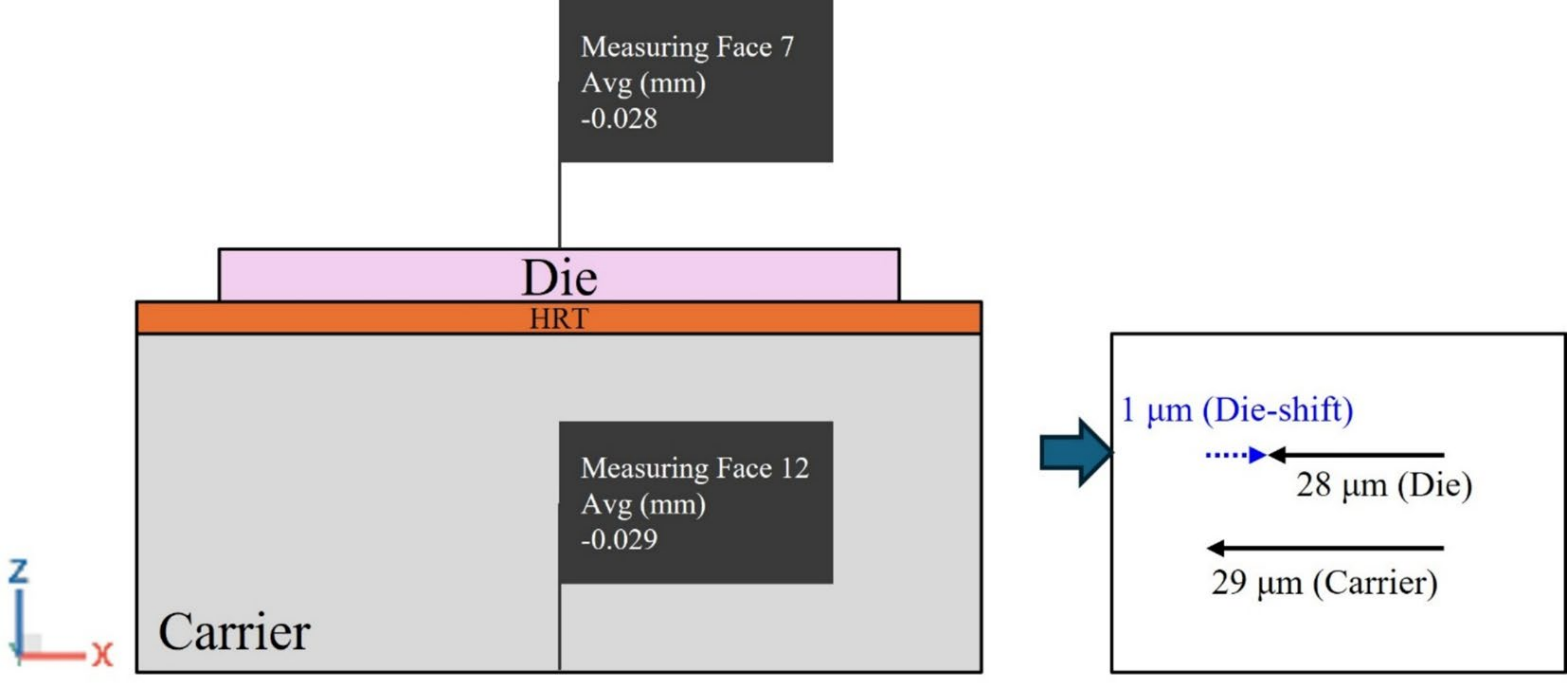
Interpretation of die shift results induced by warpage effects.

Die shift values induced by warpage effects are defined as the relative displacement between die and carrier, as illustrated in Fig. 13, which shows the X-direction displacement as an example.

**Fig. 14:**
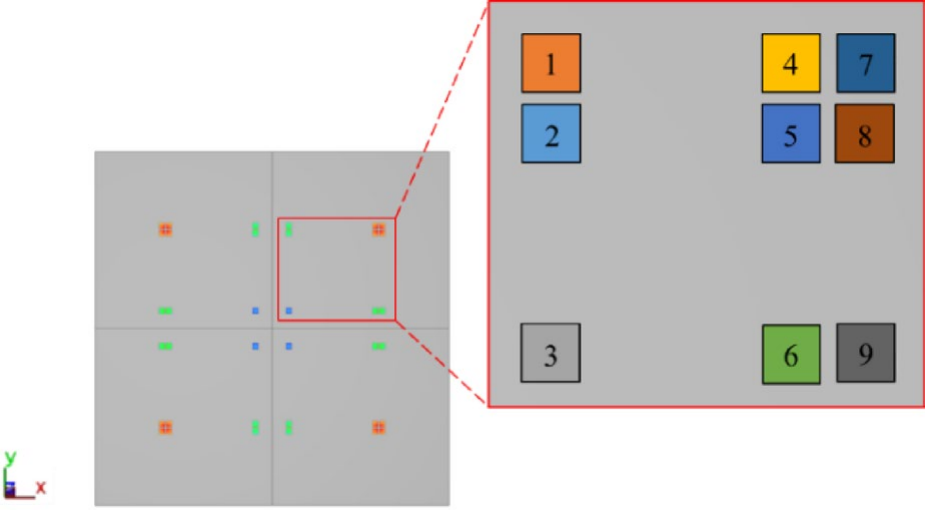
Schematic of the nine die locations.

Furthermore, given the quarter-symmetry in the die shift simulation results, the analysis is focused on only nine dies within a single sub-panel. The locations of these nine dies are illustrated in Fig. 14.

**Fig. 15:**
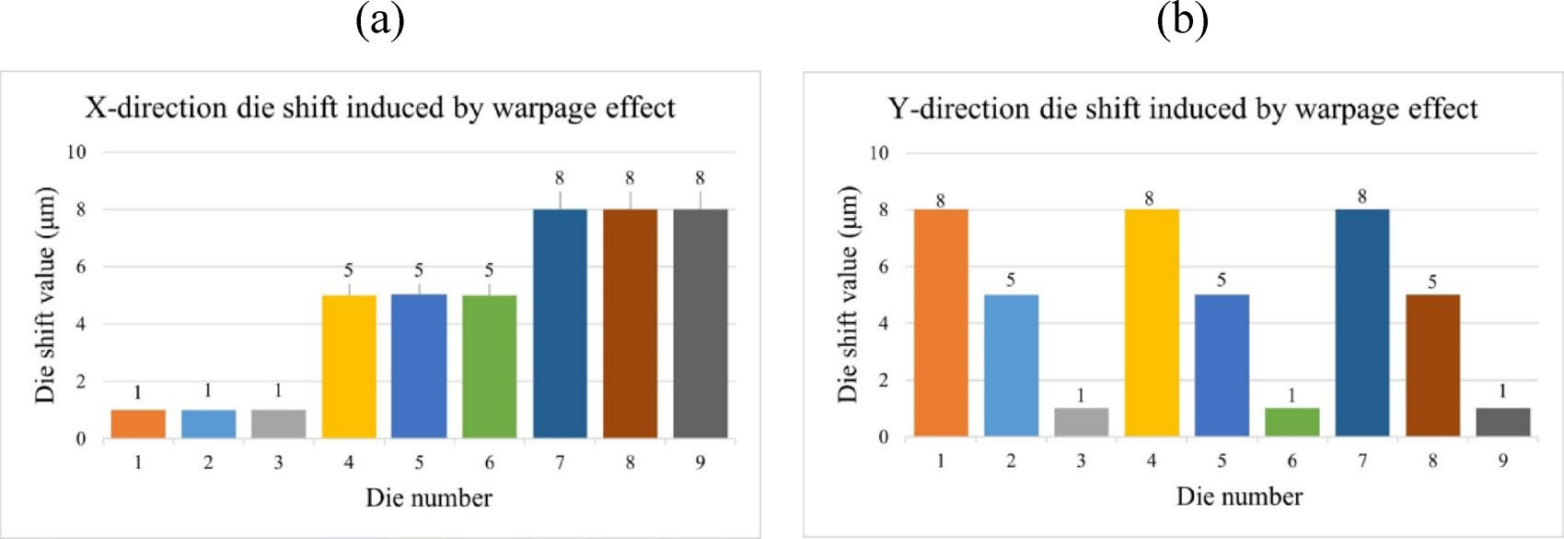
(**a**) Die shift distribution along X-direction due to warpage effect (**b**) Die shift distribution along Y-direction due to warpage effect.

Figure 15 illustrates the simulated die shift results induced by warpage effects, with Fig. 15 (a) showing the X-direction and Fig. 15 (b) the Y-direction displacements. In both directions, the maximum die shift reaches 8 μm. In the X-direction, as shown in Fig. 15 (a), the die shift gradually increases from the center toward the right edge of the panel. Dies 7, 8, and 9 exhibit the largest displacement, indicating that dies located near the outer boundary are more susceptible to warpage-induced movement. In contrast, Fig. 15 (b) shows that in the Y-direction, the highest shifts occur at dies 1, 4, and 7, which are distributed along the perimeter of the panel.

The larger displacement observed at dies 7, 8, and 9 mainly results from the boundary condition of the model. Since the panel is constrained at its center, the central region acts as the structural support point. When a mismatch in the coefficients of thermal expansion (CTE) occurs among the materials, stress concentration develops, causing the die closest to the center (die 1) to exhibit the smallest warpage, while the dies farther from the center (dies 7, 8, and 9) show the largest warpage, resulting in larger displacement.

#### Die shift results with fluid-flow effect

**Fig. 16:**
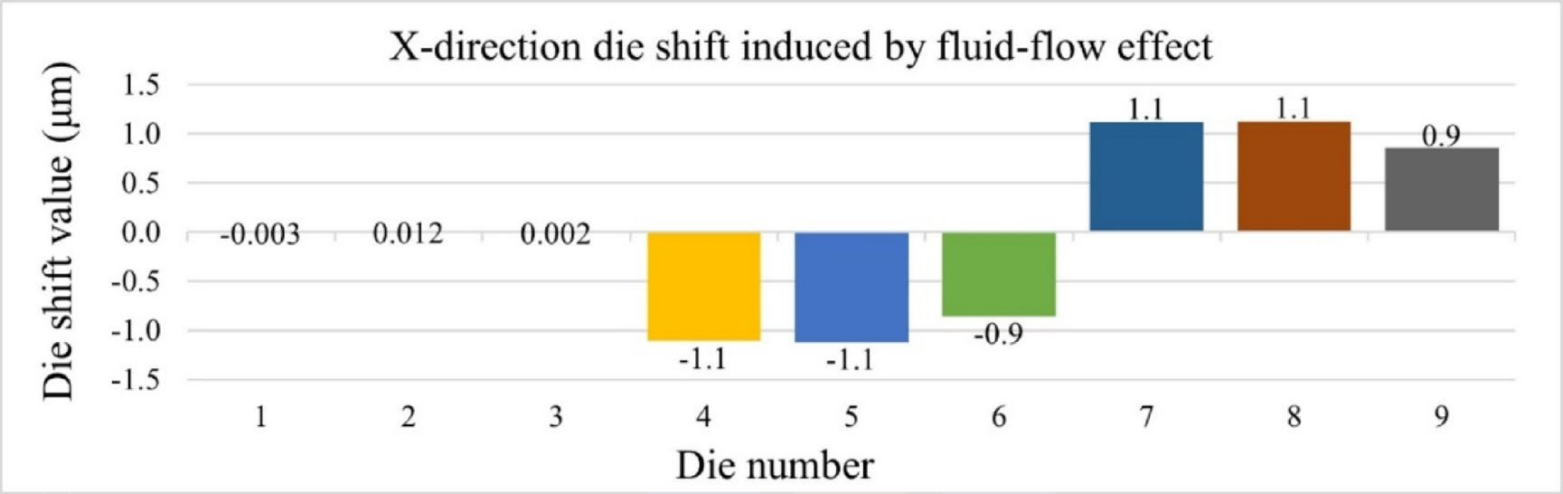
Die shift distribution along X-direction due to fluid-flow effects.

**Fig. 17:**
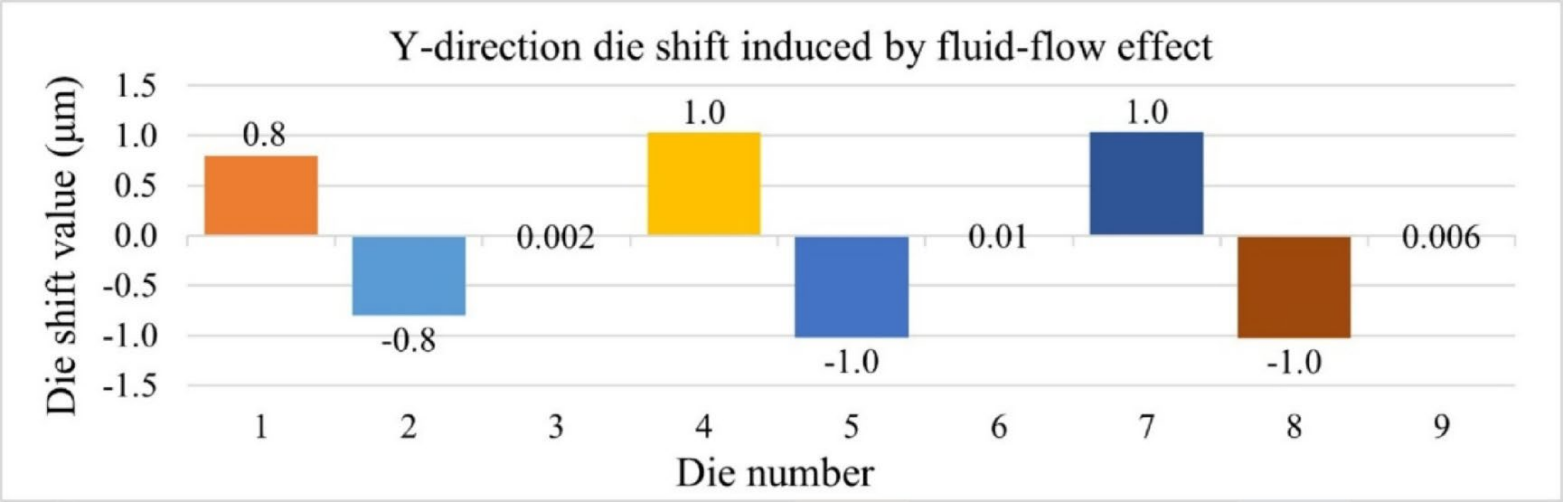
Die shift distribution along Y-direction due to fluid-flow effects.

Figures 16 and 17 present the die shift results induced by fluid-flow effects in the X and Y directions, respectively. In both directions, the largest displacements are observed at die positions 4, 5, 7, and 8, indicating that central and near-edge regions of the panel are more affected by non-uniform resin flow during compression molding. Under the assumption that the heat release tape exhibits room-temperature material properties, the maximum die shift is limited to 1.1 μm.

These results serve as a baseline and will be compared with simulations incorporating temperature-dependent HRT properties in the next section to evaluate the impact of HRT softening at elevated temperatures on fluid-induced die shift. Based on the results mentioned above, relying solely on room-temperature HRT properties could not accurately represent the actual die shift behavior. To further investigate this effect, nanoindentation experiments were conducted to evaluate the mechanical behavior of HRT at elevated temperatures. The temperature-dependent data obtained from these measurements will subsequently be integrated into simulation models for further validation against experimental observations.

## Nanoindentation experiments

In light of the possible inaccuracy associated with using room-temperature HRT properties, this section focuses on investigating the mechanical behavior of HRT at elevated temperatures through nanoindentation experiments. Nanoindentation experiments were performed to determine the Young’s modulus of both the pressure-sensitive adhesive (PSA) and the foaming adhesive layer within the HRT structure at room temperature and elevated temperatures. For simulation purposes, a three-layer HRT model was constructed using the temperature-dependent modulus values obtained from the nanoindentation tests. This refined material representation enables more accurate high temperature die shift simulations and provides a foundation for comparison with experimental measurements.

### HRT structure

The heat release tape (HRT) is a functional adhesive material commonly composed of a polyethylene terephthalate (PET) film substrate coated with a pressure-sensitive adhesive layer. It is primarily used to temporarily secure carrier substrates during wafer handling and die singulation processes. One of the key features of HRT is its significant reduction in adhesive strength at elevated temperatures, which facilitates clean and efficient debonding with minimal residue. While this thermal response enhances process efficiency during dicing and separation, it also contributes to increased die shift during the subsequent compression molding process due to the loss of structural support under high temperatures.

### Nanoindentation experiment

**Fig. 18:**
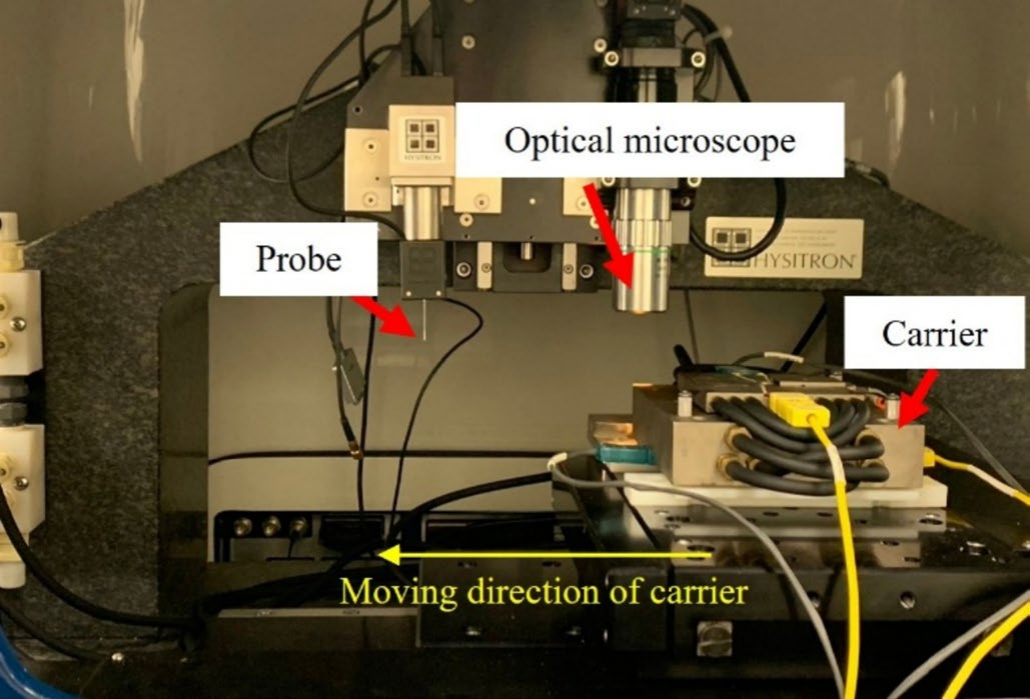
Nanoindenter.

Figure 18 shows the nanoindentation system used in this study, which is equipped with a precision optical microscope, indenter probe, and programmable carrier stage. Nanoindentation is a high-resolution mechanical characterization technique capable of measuring localized properties of thin films and microstructures, including hardness, surface roughness, and reduced Young’s modulus. The instrument is further integrated with a temperature-controlled environment, enabling reliable measurement of material properties across a wide range of temperatures. In this study, nanoindentation experiments were performed on the pressure-sensitive adhesive and foaming adhesive layers within the heat release tape to capture their temperature-dependent mechanical behavior for subsequent simulation input.

#### Experiment procedures and test conditions

**Fig. 19:**
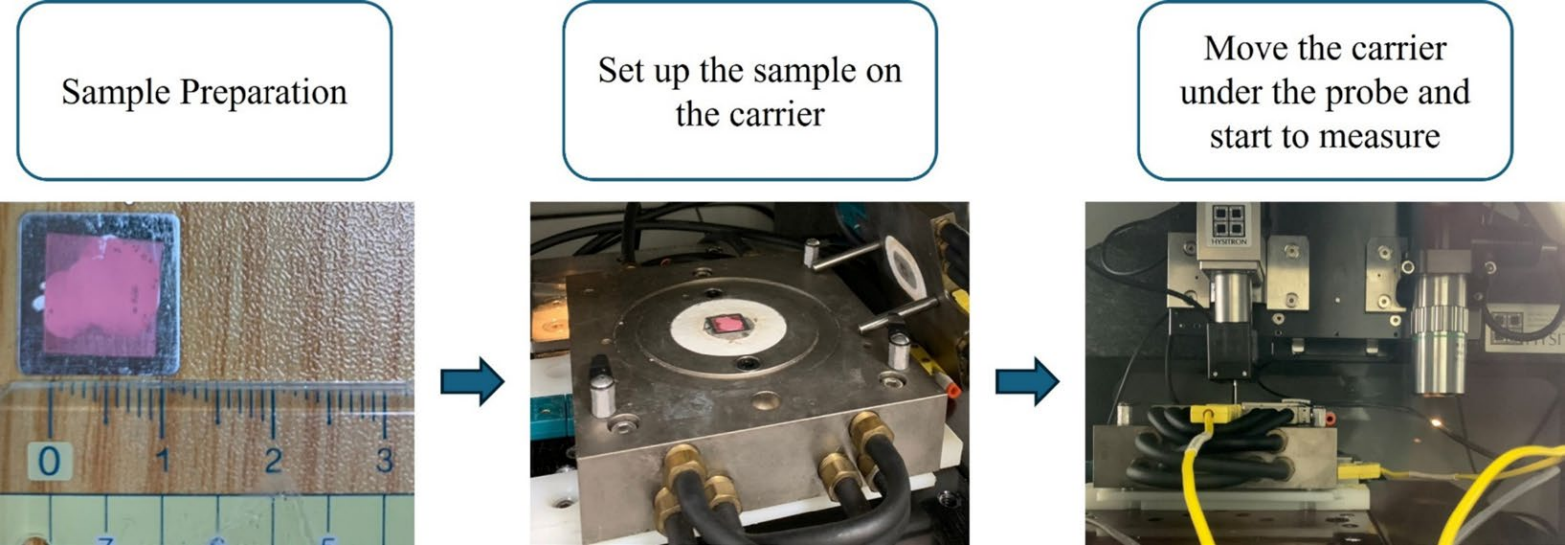
Nanoindentation experiment flow chart.

**Fig. 20:**
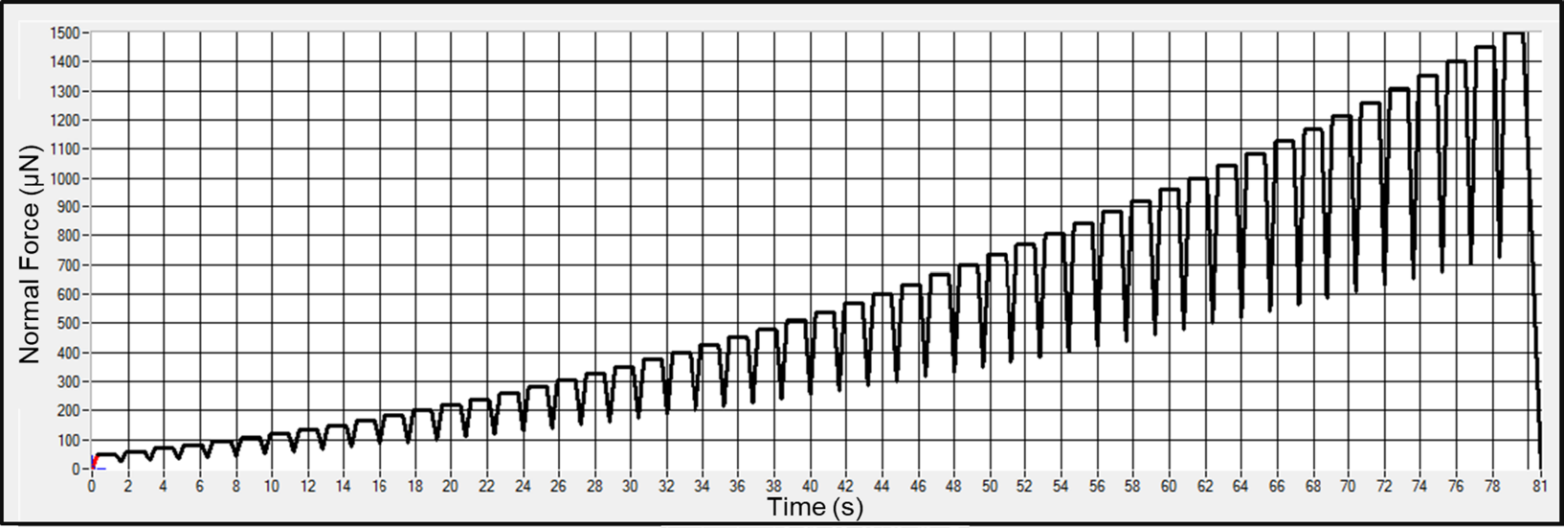
Testing condition of Nanoindenter.

The experiment procedure for nanoindentation is shown in Fig. 19. The process includes sample preparation, mounting the sample onto the heated carrier stage, and aligning it beneath the indenter probe for measurement. Each indentation test was conducted at a fixed point using 50 loading-unloading cycles to assess the mechanical response under repeated load application. The applied load ranged from 50 to 1500 µN, as shown in the loading profile in Fig. 20. This stepped-loading approach enables the observation of depth-dependent mechanical behavior at a single location, providing greater resolution in evaluating the material’s modulus variation with indentation cycles.

#### Results of nanoindentation experiment

**Fig. 21:**
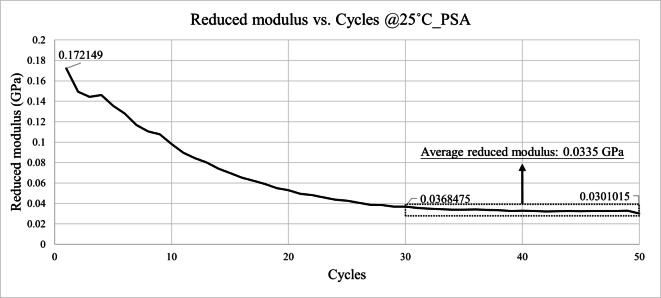
Reduced modulus results of PSA @25˚C.

**Fig. 22:**
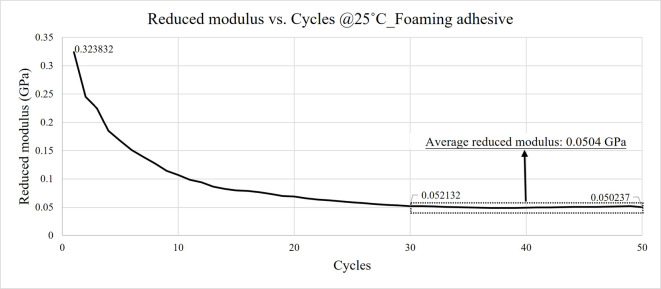
Reduced modulus results of foaming adhesive @25˚C.

Figures 21 and 22 illustrate the variation in reduced modulus with indentation depth for the two constituent layers of the HRT material at room temperature. The reduced modulus values were observed to stabilize between cycles 30 and 50 across different depths. To minimize the influence of surface oxidation and contact area uncertainty, the average modulus values from cycles 30 to 50 were extracted for subsequent analysis.

**Fig. 23:**
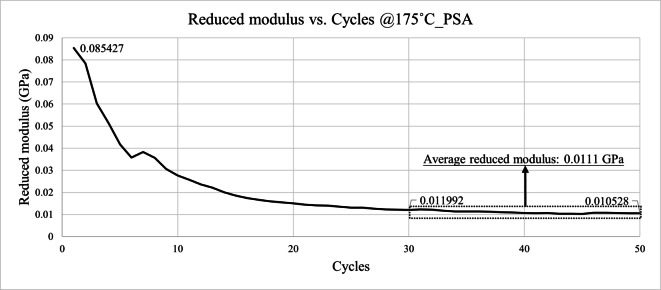
Reduced modulus results of PSA @175˚C.

**Fig. 24:**
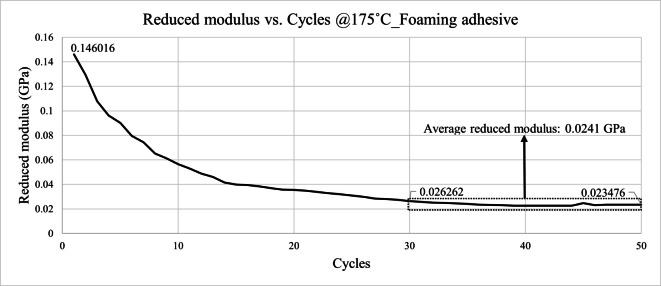
Reduced modulus results of foaming adhesive @175˚C.

Figures 23 and 24 present the reduced modulus results obtained at 175 °C. The results indicate that both the pressure-sensitive adhesive and foaming adhesive layers exhibited significant softening at elevated temperatures, with their reduced modulus decreasing to approximately one-third to one-half of the values measured at room temperature. This temperature-dependent degradation in mechanical stiffness confirms the necessity of incorporating high-temperature material properties into the die shift simulation.

A gradual decrease in reduced modulus was observed with increasing indentation cycles, as shown in Figs. 21, 22, 23 and 24. This behavior can be attributed to progressive plastic deformation and viscoelastic relaxation occurring within the adhesive layers under repeated loading. Similar trends have been reported in previous cyclic nanoindentation studies. Fang et al. investigated semiconductor and metal thin films and found that both hardness and Young’s modulus decreased with increasing number of indentation cycles due to dislocation accumulation, increase of contact area, and continuous local plastic deformation^18^.

#### Relationship between reduced modulus and young’s modulus

To account for potential deformation of the nanoindenter probe during the nanoindentation process, the reduced modulus values obtained from the experiments were converted into the corresponding Young’s modulus of the sample using the method proposed by Shuman^19^.

16 $$\:\frac{\mathrm{1}}{\mathrm{E}\mathrm{*}}\text{}\mathrm{=}\:\frac{\mathrm{(1}\mathrm{-}\mathrm{v}\mathrm{s}\mathrm{2}\mathrm{)}}{\mathrm{E}\mathrm{s}}\text{}\mathrm{+\:}\frac{\mathrm{(1}\mathrm{-}\mathrm{v}\mathrm{i}\mathrm{2}\mathrm{)}}{\mathrm{E}\mathrm{i}}$$

$\:\mathrm{E}\mathrm{*}$: Reduced modulus; $\:\mathrm{E}\mathrm{s}$: Young’s modulus of sample; $\:\mathrm{v}\mathrm{s}$: Poisson’s ratio of sample; $\:\mathrm{E}\mathrm{i}$: Young’s modulus of probe; $\:\mathrm{v}\mathrm{i}$: Poisson’s ratio of probe.

The calculated Young’s modulus values for the HRT layers are summarized in Table 3. At 25 °C, the pressure-sensitive adhesive and foaming adhesive exhibit Young’s modulus of 24.6 MPa and 38.3 MPa, respectively. At an elevated temperature of 175 °C, both materials show a significant reduction in stiffness, with Young’s modulus decreasing by approximately 50 to 65%, corresponding to values of 8.4 MPa and 18.3 MPa.

Due to the limited indentation depth, direct measurement of the PET substrate was not feasible. Therefore, its mechanical properties were referenced from Chen^20^, which reports the Young’s modulus of optoelectrical-grade PET films as 190 MPa at room temperature. The high-temperature Young’s modulus was estimated using the same softening ratio observed in the PSA and foaming adhesive layers. As both adhesive materials showed a reduction to approximately one-third to one-half of their room-temperature modulus when heated to 175 °C, the PET modulus was proportionally reduced to one-third of its room-temperature value, resulting in 63.3 MPa.

In addition, the experimentally obtained Young’s modulus values for PSA and foaming adhesive were combined using the rule of mixtures to estimate the effective Young’s modulus of the full HRT structure at room temperature. These results were compared with the modulus values provided by Innolux Corporation, as listed in Table 4, to validate the accuracy of the nanoindentation-based characterization.

**Table 3:** Young’s modulus of HRT material at different temperatures.

Temperature Material	Room temperature @25˚C	High-temperature @175˚C
PSA	24.6	8.4
Foaming adhesive	38.3	18.3
PET film	190	63.6

**Table 4:** Nanoindentation validation data at room temperature.

	Young’s Modulus (MPa)
Provided by Innolux	108
Experiment by rule of mixture	96.9

### Die shift analysis with high temperature HRT properties

Based on the die shift results induced by the mold flow effect discussed in Sect. 3.1.5, the maximum displacement was limited to 1 μm. This underestimation is primarily attributed to the neglect of heat release tape (HRT) softening behavior at elevated temperatures in the previous simulation. To address this limitation, the current section evaluates the influence of high-temperature HRT properties on die shift behavior by incorporating temperature-dependent mechanical characteristics obtained through nanoindentation.

**Fig. 25:**
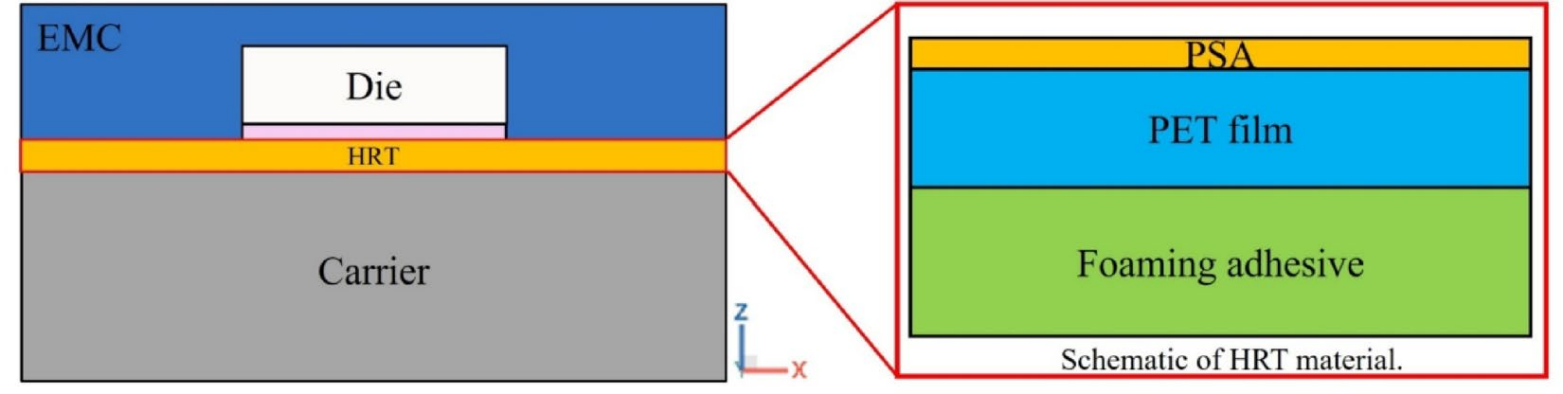
Schematic sectional view of the model with three-layer structure of HRT.

The geometric model used in this analysis is consistent with that described in Sect. 3.1 (refer to Figs. 8 and 9). The main difference lies in the implementation of a three-layer HRT structure, comprising PET film, pressure-sensitive adhesive (PSA), and foaming adhesive, as illustrated in Fig. 25. The Young’s modulus values for the PSA and foaming adhesive were taken from the nanoindentation results presented in Sect. 4.2.

#### Die shift results with warpage effect

**Fig. 26:**
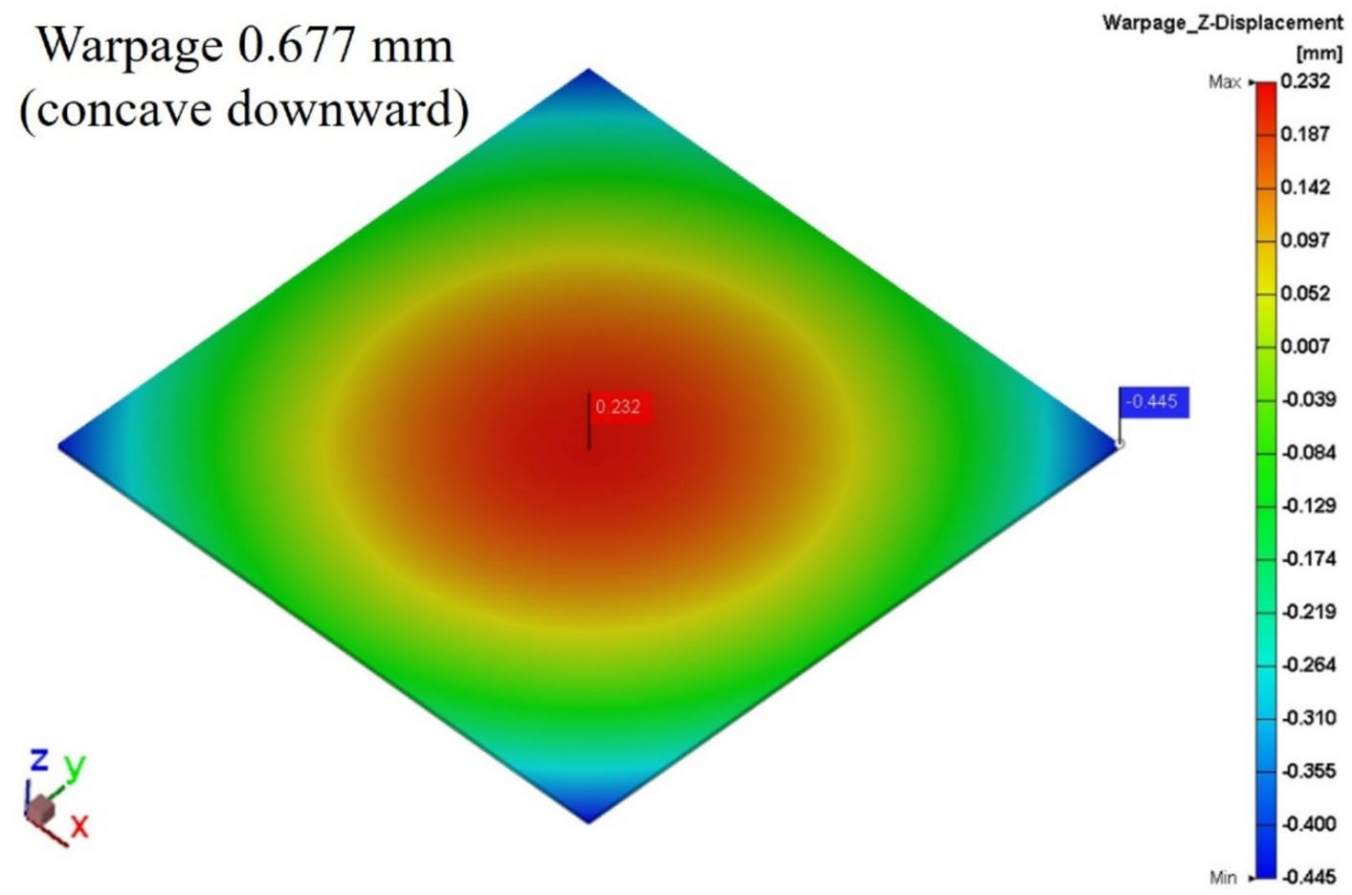
Die shift result of single layer HRT induced by warpage effect.

**Fig. 27:**
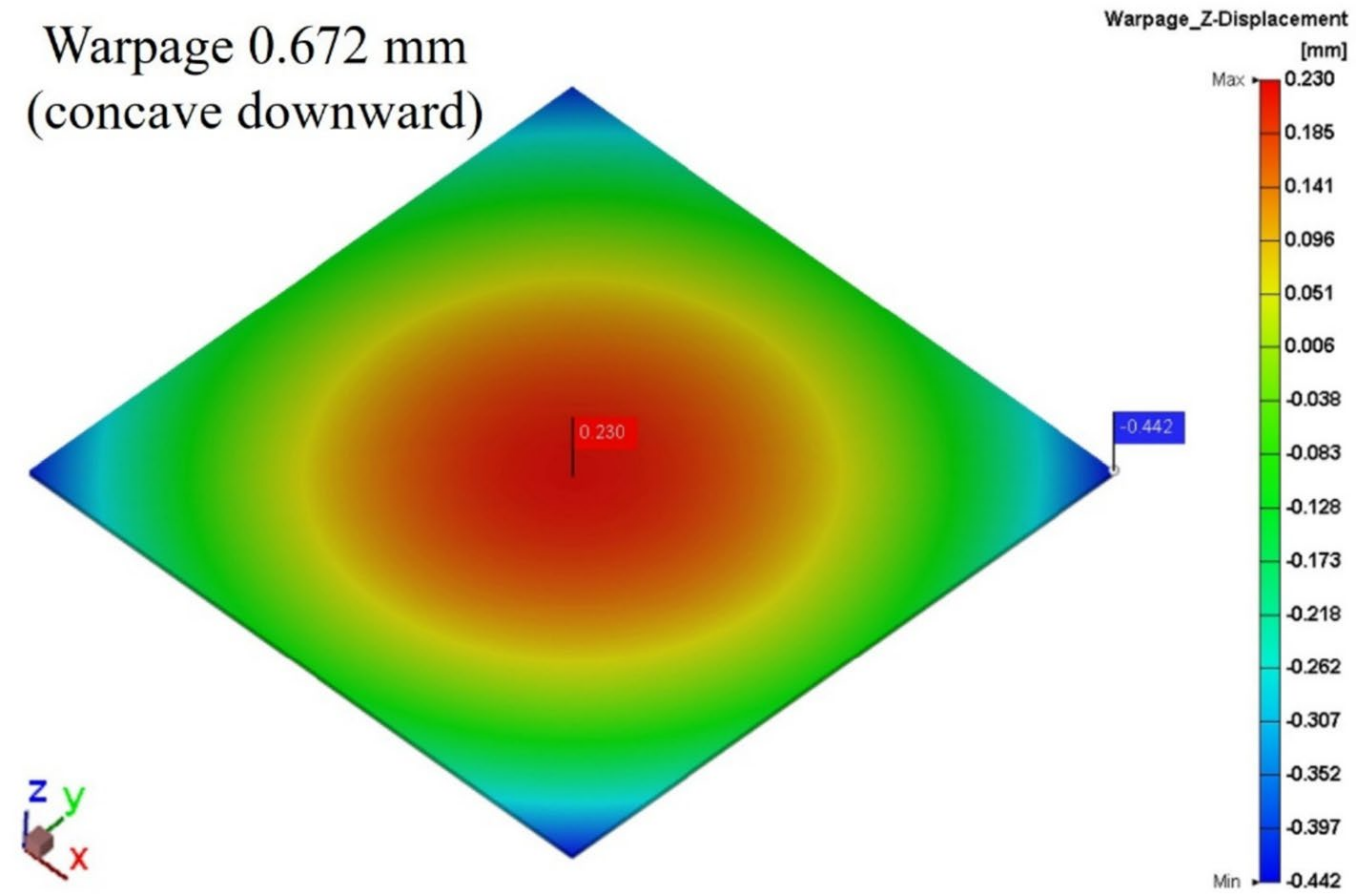
Die shift result of three-layer HRT induced by warpage effect.

Figures 26 and 27 present the warpage simulation results for the package modeled using a single-layer HRT structure and a three-layer HRT structure, respectively. The warpage distributions in both cases are highly similar, indicating that the HRT region has negligible influence on the overall package deformation.

Furthermore, the equivalent Young’s modulus calculated at room temperature was 96.9 MPa, close to the reference value of 108 MPa provided by Innolux Corporation. This consistency confirms the validity of the three-layer HRT modeling approach and indicates that internal structural variation within the HRT has minimal impact on global warpage behavior. Consequently, the die shift induced by warpage remains nearly identical across both modeling configurations.

#### Die shift results with Fluid-flow effect

**Fig. 28:**
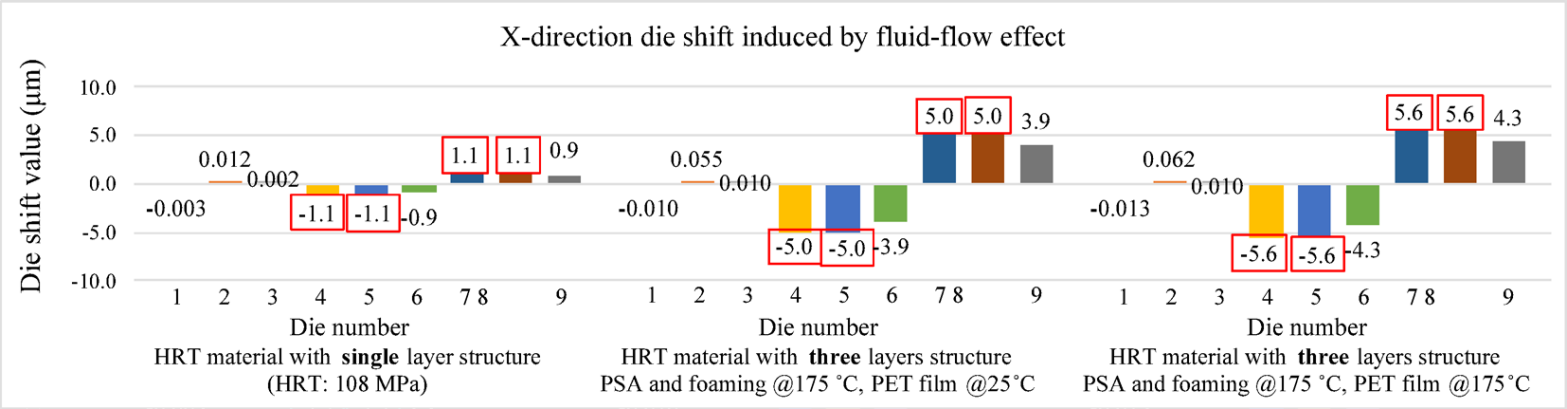
Die shift results induced by fluid-flow effects (X-direction).

**Fig. 29:**
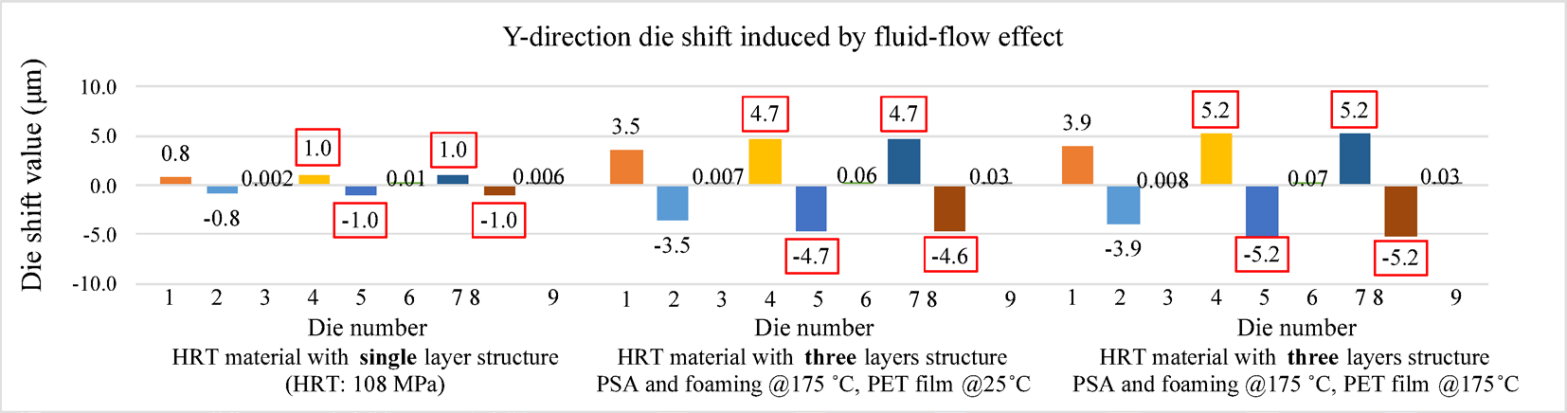
Die shift results induced by fluid-flow effects (Y-direction).

Figures 28 and 29 present the simulated die shift results caused by mold flow effects under three different modeling conditions. In the first case, the Young’s modulus of the heat release tape was assumed to remain at room temperature. The second case incorporated the high-temperature Young’s modulus for the PSA and foaming adhesive layers, while retaining the room-temperature value for the PET film. In the third case, the Young’s modulus of the entire heat release tape structure was defined according to elevated temperature conditions.

In all three cases, pronounced die shifts were observed at die positions 4, 5, 7, and 8. This concentration of displacement is likely attributed to the denser geometric arrangement in these areas, as illustrated in the die layout of the panel shown in Fig. 12. The results reveal a clear amplification of mold-flow-induced die shift when high-temperature material properties are applied.

The observed increase in die shift with decreasing HRT Young’s modulus can be explained by the interfacial shear transfer mechanism. The HRT serves as an interlayer between the carrier and the EMC. A lower Young’s modulus reduces the interfacial constraint and shear stiffness, allowing greater lateral movement of the dies during cooling, as demonstrated in the second and third cases. The similarity between these two cases indicates that the PET film, which is located between the PSA and the foaming adhesive tape, has minimal influence on die shift behavior, owing to its relatively stable mechanical properties at elevated temperatures.

#### Total die shift results and experiment results

As summarized in Table 5, the total die shift at high temperature was obtained by vector summation of the warpage- and flow-induced components. For instance, Die 5 exhibited a displacement of + 4.9 μm caused by warpage and − 5.6 μm caused by mold flow in the X-direction, resulting in a net displacement of −0.7 μm. This result indicates that the opposing deformation directions of the two mechanisms can locally offset each other, leading to a reduced overall die shift.

**Table 5:** Comparison of the total die-shift simulation result for high temperature.

	Warpage (µm)		Fluid-flow (µm)		Combined effects (µm)		
	X	Y	X	Y	X	Y	Total
1	1.2	7.6	−0.01	3.9	1.19	11.5	11.6
2	1.2	4.7	0.06	−3.9	1.26	0.8	1.5
3	1.1	1.2	0.01	0.01	1.11	1.21	1.6
4	4.9	8.0	−5.6	5.2	−0.7	13.2	13.2
5	4.9	4.4	−5.6	−5.2	−0.7	−0.8	1.1
6	5.1	1.3	−4.3	0.07	0.8	1.37	1.6
7	8.3	8.0	5.6	5.2	13.9	13.2	19.2
8	8.3	4.4	5.6	−5.2	13.9	−0.8	13.9
9	7.9	1.3	4.3	0.03	12.2	1.33	12.3

**Fig. 30:**
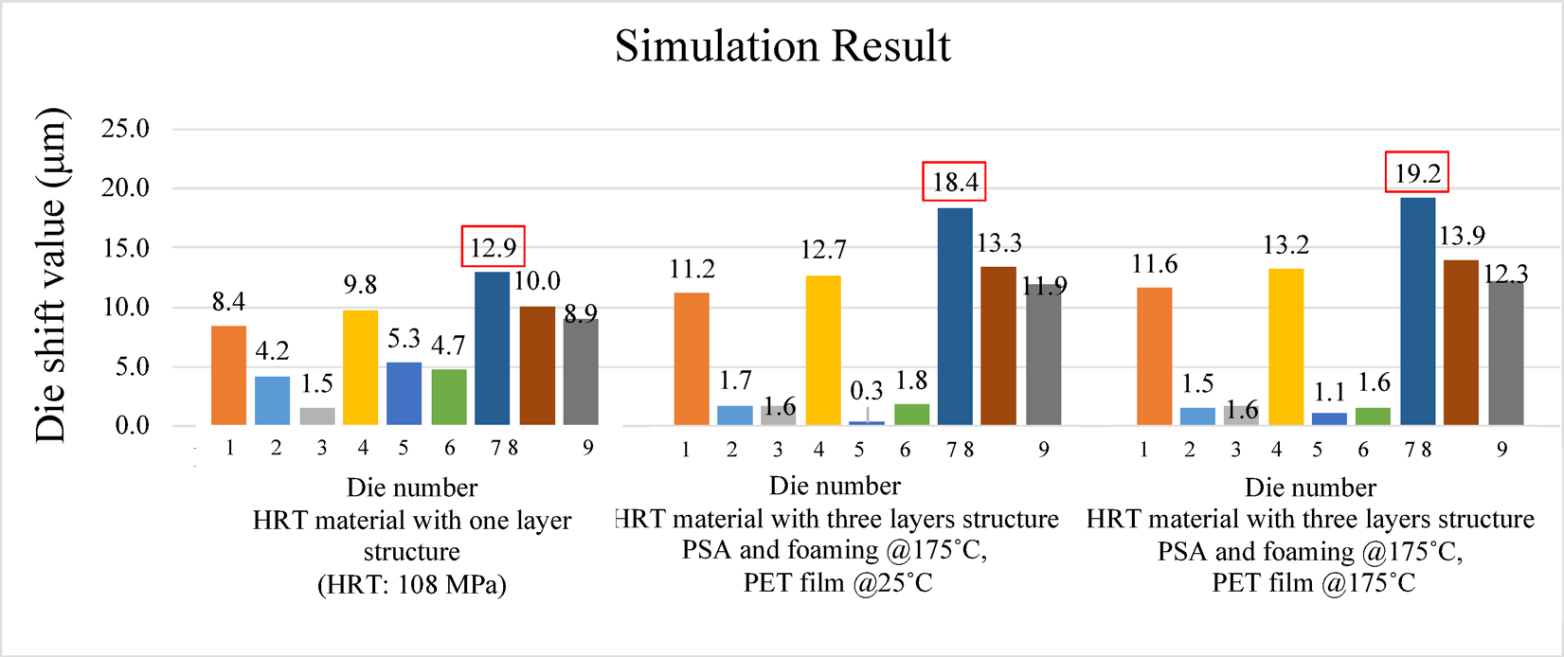
Comparison of total die-shift simulation results under various conditions.

Figure 30 illustrates the total die shift simulation results, which combine the effects of mold flow and warpage under three different material conditions. These include using the Young’s modulus of the heat release tape at room temperature, applying high-temperature modulus values for the pressure-sensitive adhesive and foaming adhesive while retaining the room-temperature modulus for the PET film, and fully adopting the high-temperature modulus for the entire heat release tape structure. The results demonstrate that incorporating the softening behavior of the heat release tape at elevated temperatures leads to a notable increase in die shift. In all cases, the maximum displacement occurs at die position 7, and the overall die shift magnitude increases with distance from the panel center. Position 5 exhibits a minimal total shift due to the opposing contributions of warpage-induced and flow-induced displacements.

**Fig. 31:**
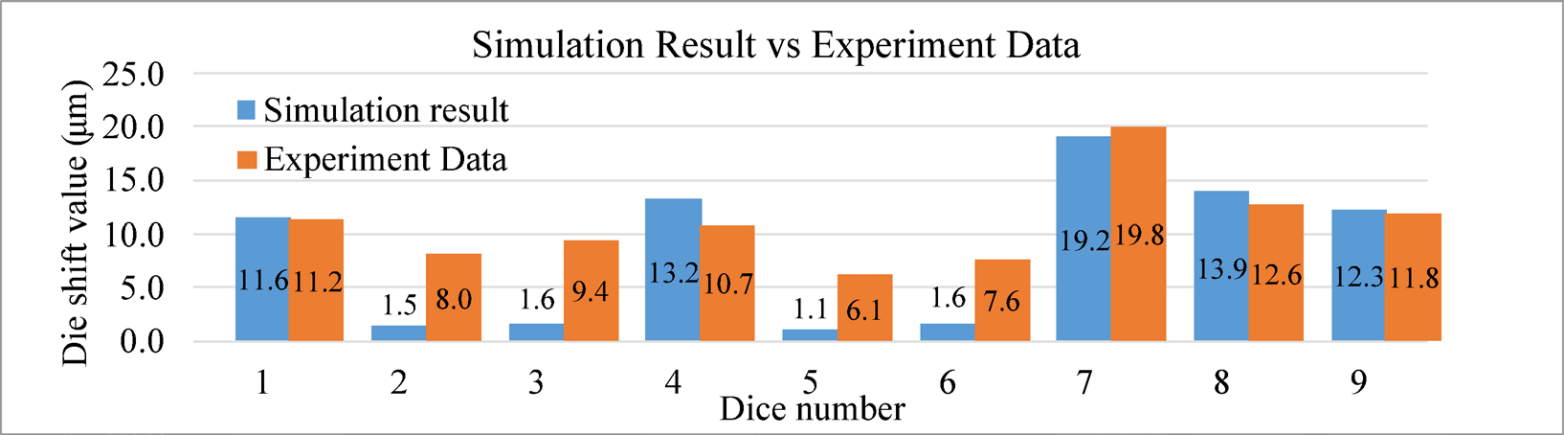
Comparison of total die shift simulation results with experimental data.

Figure 31 presents a comparison between the simulation results and the experimental measurements provided by Innolux Corporation. The simulation values combine the displacement caused by warpage and fluid flow. Notably, the results at die positions 1, 4, 7, 8, and 9 show good agreement with the experimental data.

From the simulation results, it can be observed that the warpage-induced displacement increases with the distance from the panel center, whereas the flow-induced displacement becomes more pronounced in regions where the surrounding dies are more densely arranged. In some locations, these two effects act in opposite directions and partially cancel each other out.

Although larger discrepancies are observed between the simulated and measured die-shift values at die positions 2, 3, 5, and 6, the overall deformation trend remains consistent across all dies. These deviations are likely caused by limited experimental data and measurement instability during the die-shift test.

## Conclusions

Die shift analysis for molding-first structures in fan-out panel-level packaging was performed using Moldex3D, with detailed consideration of the compression molding process parameters and the advanced material behavior of epoxy molding compounds, including viscosity, cure kinetics, and *P*-*V*-*T*-*C* models. Thermal and chemical shrinkage were also incorporated to ensure a physically accurate simulation framework.

A key highlight of this study is the integration of high-temperature mechanical properties of heat release tape into the simulation model. Nanoindentation experiments were conducted to measure the Young’s modulus of pressure-sensitive adhesive and foaming adhesive layers across temperature ranges. Compared with the reference values provided by Innolux Corporation at 25 °C, a deviation of 10.2% was observed. The measured results confirmed that the Young’s modulus of HRT decreases significantly at elevated temperatures, by approximately two to three times.

Die shift simulations incorporating both fluid-flow and warpage effects revealed that warpage remains the dominant mechanism. This is attributed to the shorter flow distances in compression molding, which limit the impact of resin-induced displacement compared to transfer molding processes. A clear spatial trend was also identified: warpage-induced die shift increased with distance from the panel center, whereas flow-induced shift became more significant in areas with dense die placement. This emphasizes the importance of coupling geometric and process parameters in predictive modeling.

When the temperature-dependent properties of the HRT were included, the maximum die shift caused by fluid-flow effects increased from 1.1 μm to 5.5 μm. The total predicted die shift, combining both effects, increased from 12.9 μm to 19.2 μm, yielding much closer agreement with experimental measurements.

In conclusion, although warpage remains the primary factor contributing to die shift in FOPLP, neglecting the temperature-dependent mechanical behavior of bonding materials such as heat release tape may result in considerable underestimation of die displacement, particularly in regions affected by fluid-induced flow. This study emphasizes the importance of integrating experimentally measured mechanical properties at elevated temperatures into the simulation process. Through the application of nanoindentation, this research accurately captures the mechanical response of pressure-sensitive and foaming adhesive components within the heat release tape structure. By incorporating these results into finite element simulations, the model exhibits a much closer alignment with experimental measurements. Overall, the findings contribute meaningful insights into the advancement of reliable and predictive modeling for next-generation semiconductor packaging technologies.

## Data Availability

Due to confidentiality agreements, the warpage measurement data cannot be publicly released. Other datasets used and analyzed during the current study are available from the corresponding author on reasonable request.
